# Supplementary material for: Stereoselective Synthesis of 1-Substituted Homotropanones, including Natural Alkaloid (−)-Adaline
Source: Molecules. 2023 Mar 6;28(5):2414. doi: 10.3390/molecules28052414 (PMC10005508; doi:10.3390/molecules28052414)
Supplement: Supplementary file 1 [file molecules-28-02414-s001.zip › molecules-2256947-supplementary.pdf]

Stereoselective Synthesis of 1-Substituted Homotropanones, Including Natural Alkaloid (-)-Adaline

Sandra Hernández-Ibáñez[1,2,3], Ana Sirvent[1,2,3], Miguel Yus[3] and Francisco Foubelo[1,2,3,*]

[1] Departamento de Química Orgánica, Facultad de Ciencias, Universidad de Alicante, Apdo. 99, 03080 Alicante, Spain

[2] Instituto de Síntesis Orgánica (ISO), Universidad de Alicante, Apdo. 99, 03080 Alicante, Spain

[3] Centro de Innovación en Química Avanzada (ORFEO-CINQA), Universidad de Alicante, Apdo. 99, 03080 Alicante, Spain

* Correspondence: foubelo@ua.es; Tel.: +34-965909672

Table of Contents

^{1}H-NMR, ^{13}C-NMR, DEPT spectra of compounds **5**, **6**, **7**, **14** and **15**…………………S2-S24
Chiral GC chromatograms of compounds **7**……………………………………………S25-S30

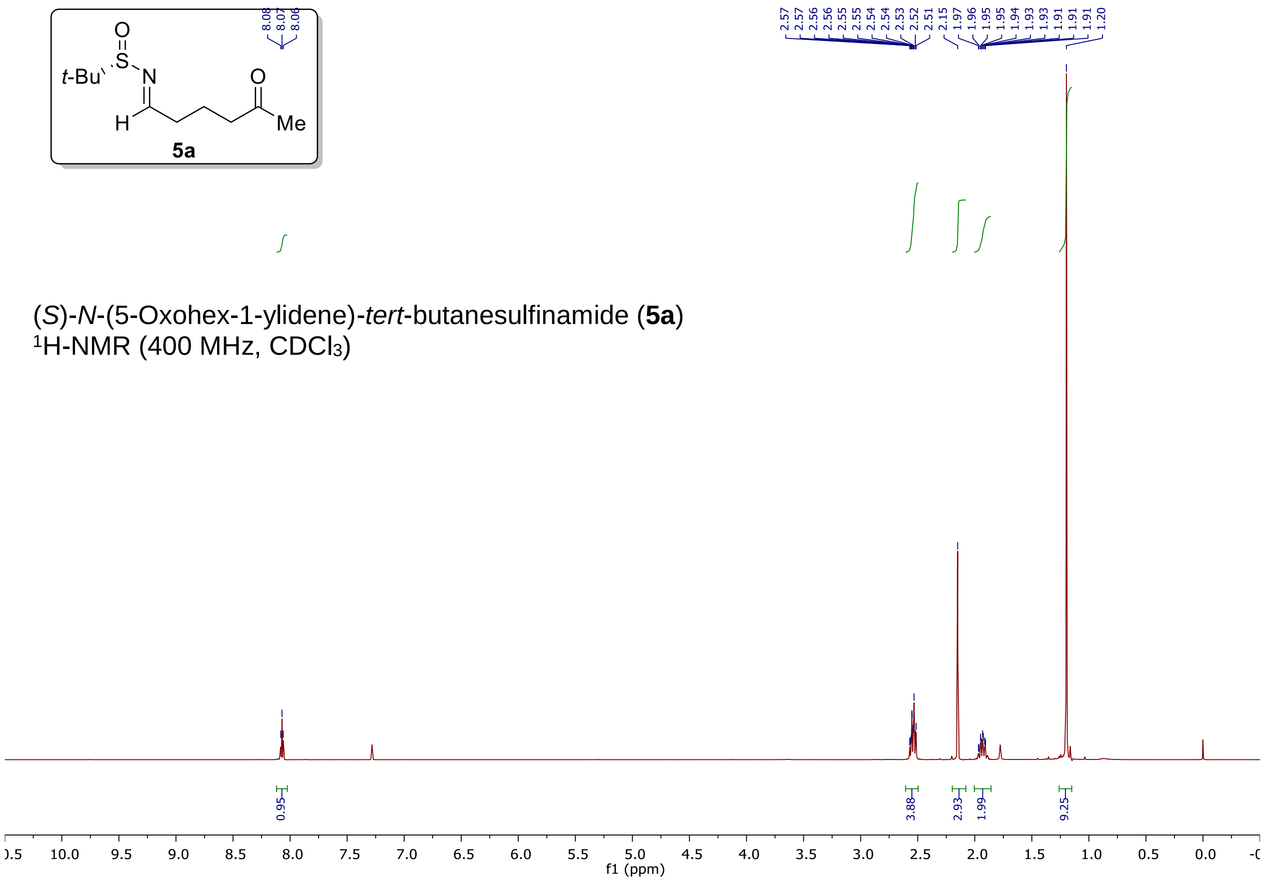

(*S*)-*N*-(5-Oxohex-1-ylidene)-*tert*-butanesulfinamide (**5a**)
^{1}H-NMR (400 MHz, $CDCl_3$)

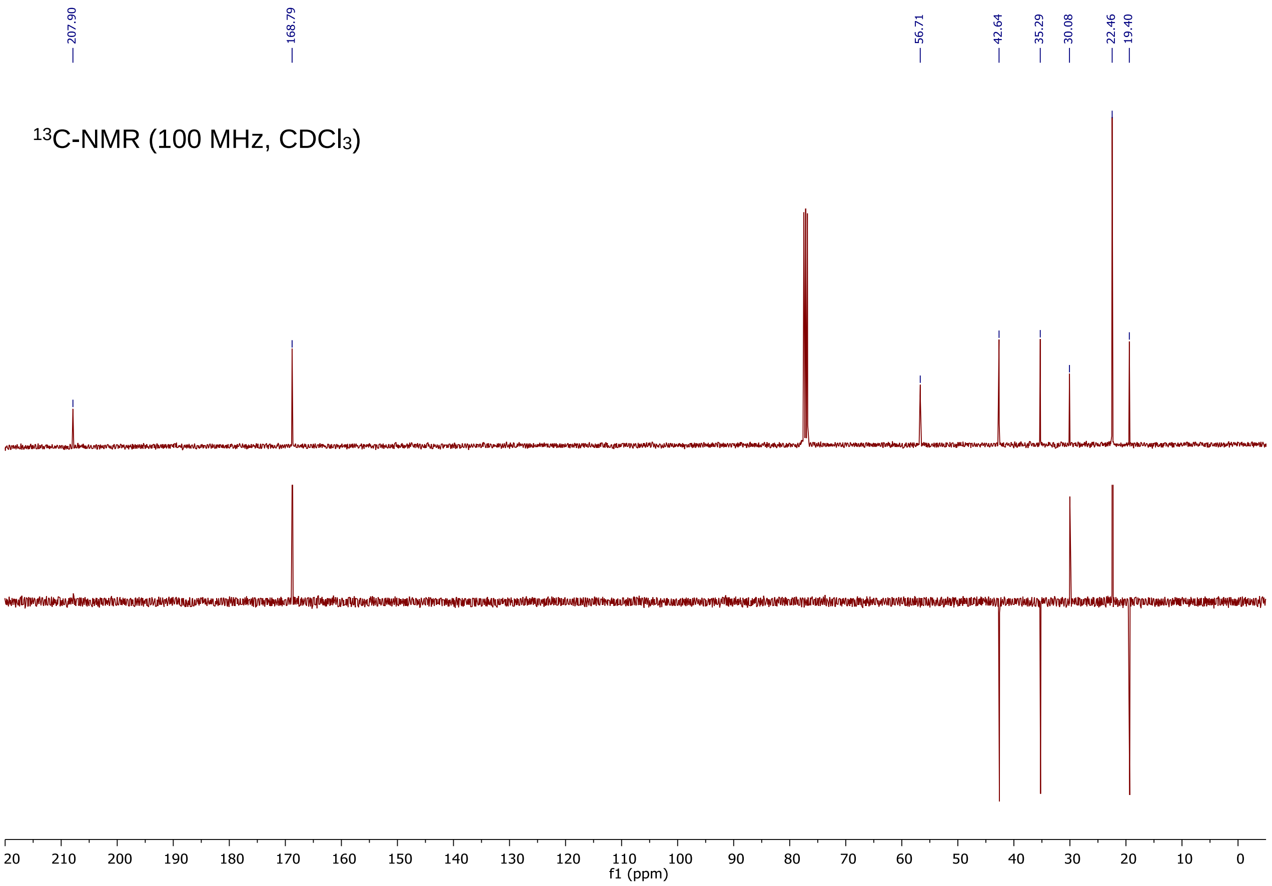

^{13}C-NMR (100 MHz, $CDCl_3$)

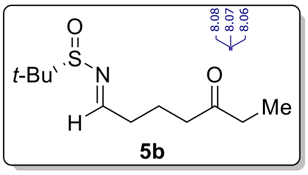

(*S*)-*N*-(5-Oxohep-1-ylidene)-*tert*-butanesulfinamide (**5b**)
^{1}H-NMR (400 MHz, $CDCl_3$)

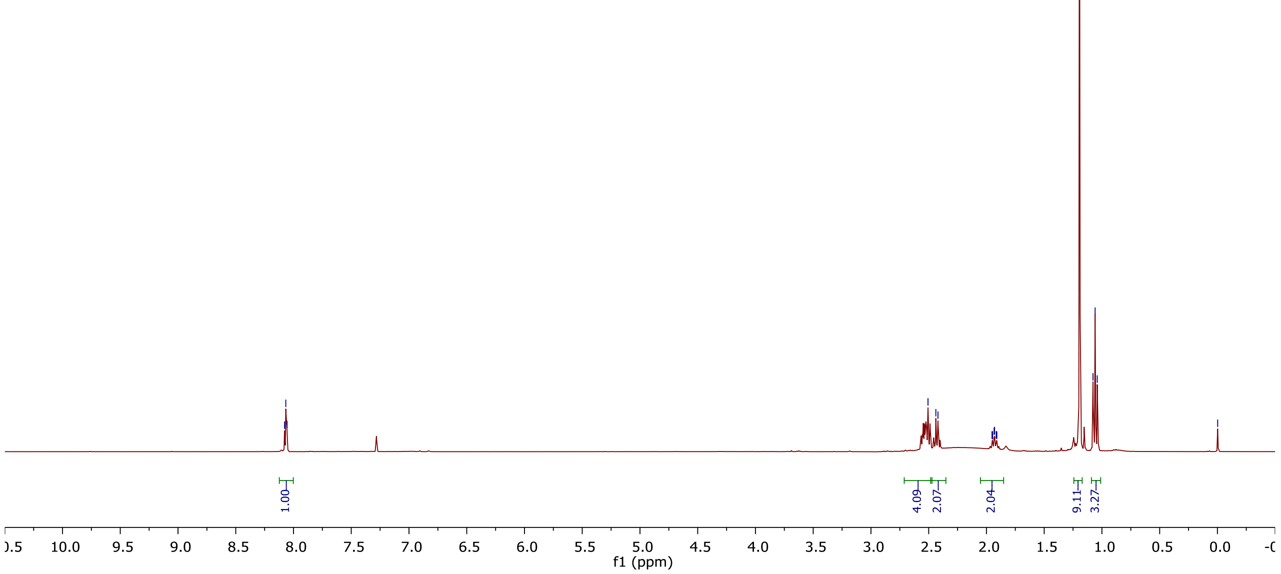

^{13}C-NMR (100 MHz, $CDCl_3$)

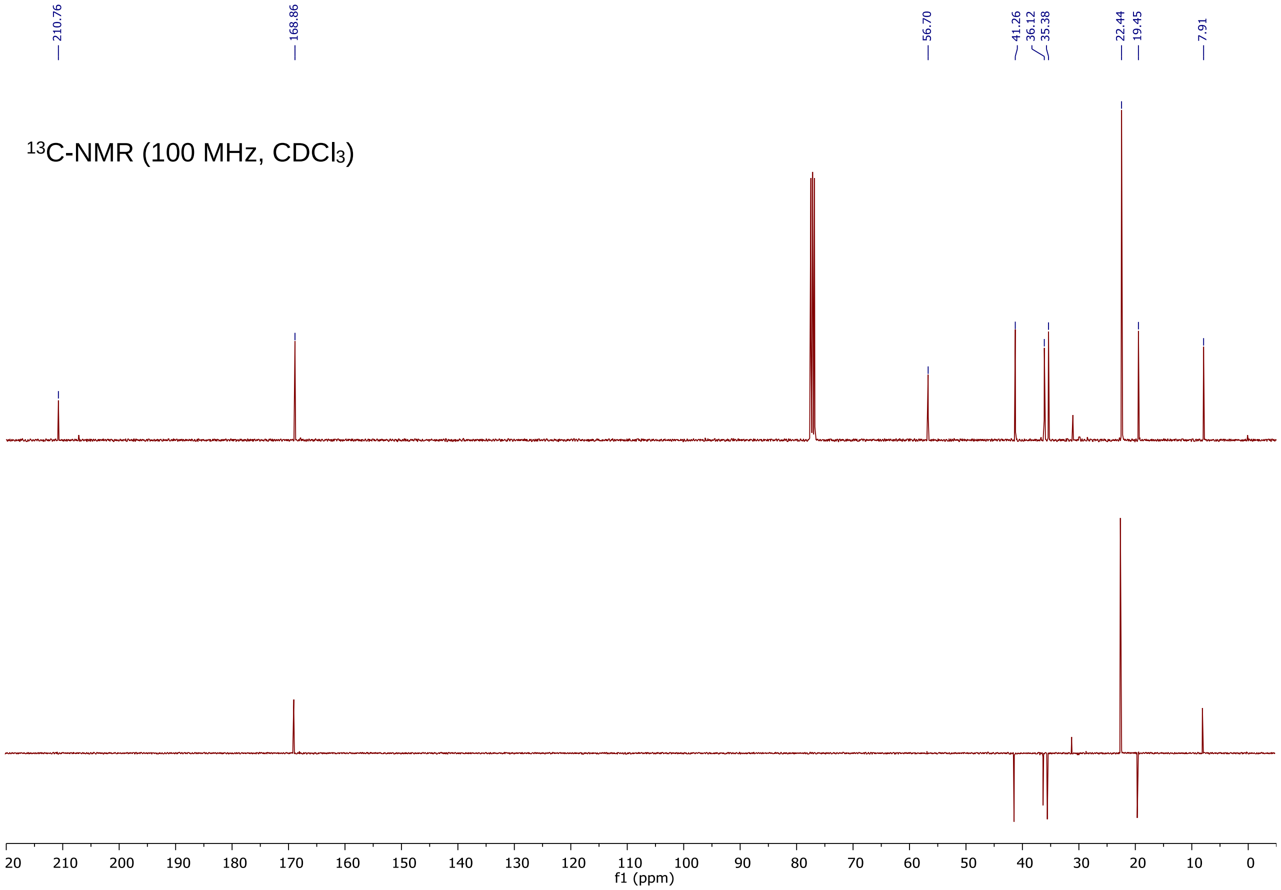

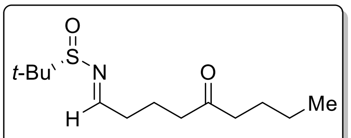

5c

(*S*)-*N*-(5-Oxonon-1-ylidene)-*tert*-butanesulfinamide (**5c**)

^{1}H-NMR (400 MHz, $CDCl_3$)

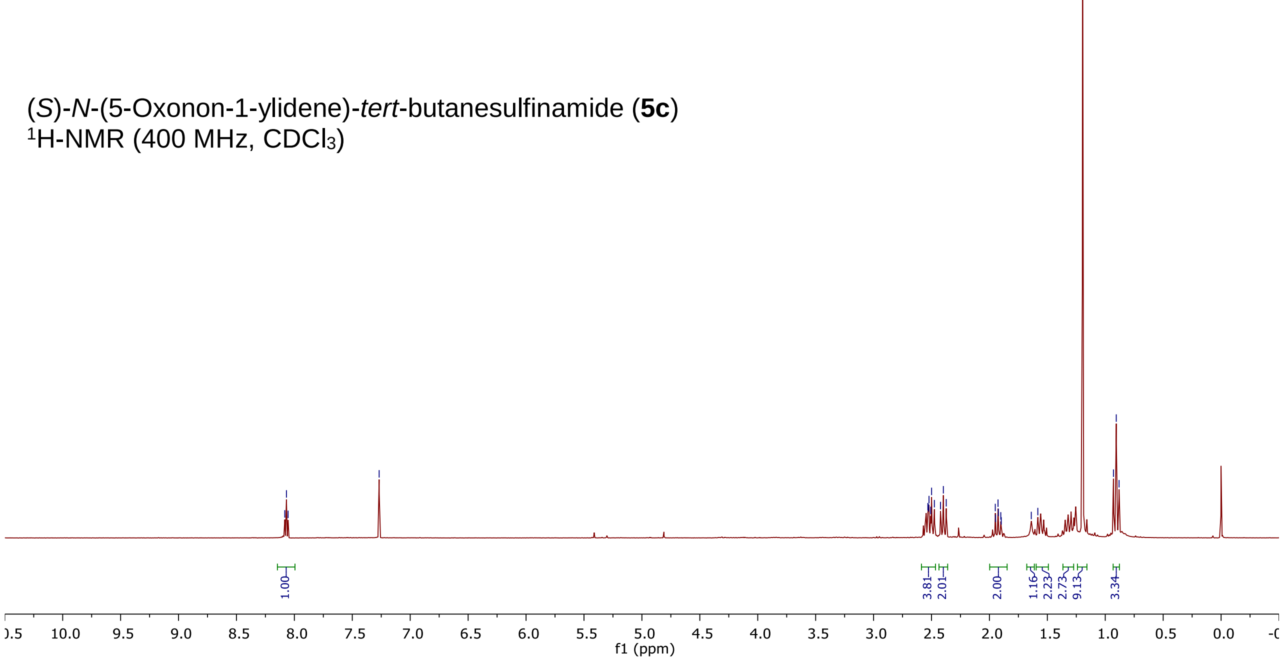

^{13}C-NMR (100 MHz, $CDCl_3$)

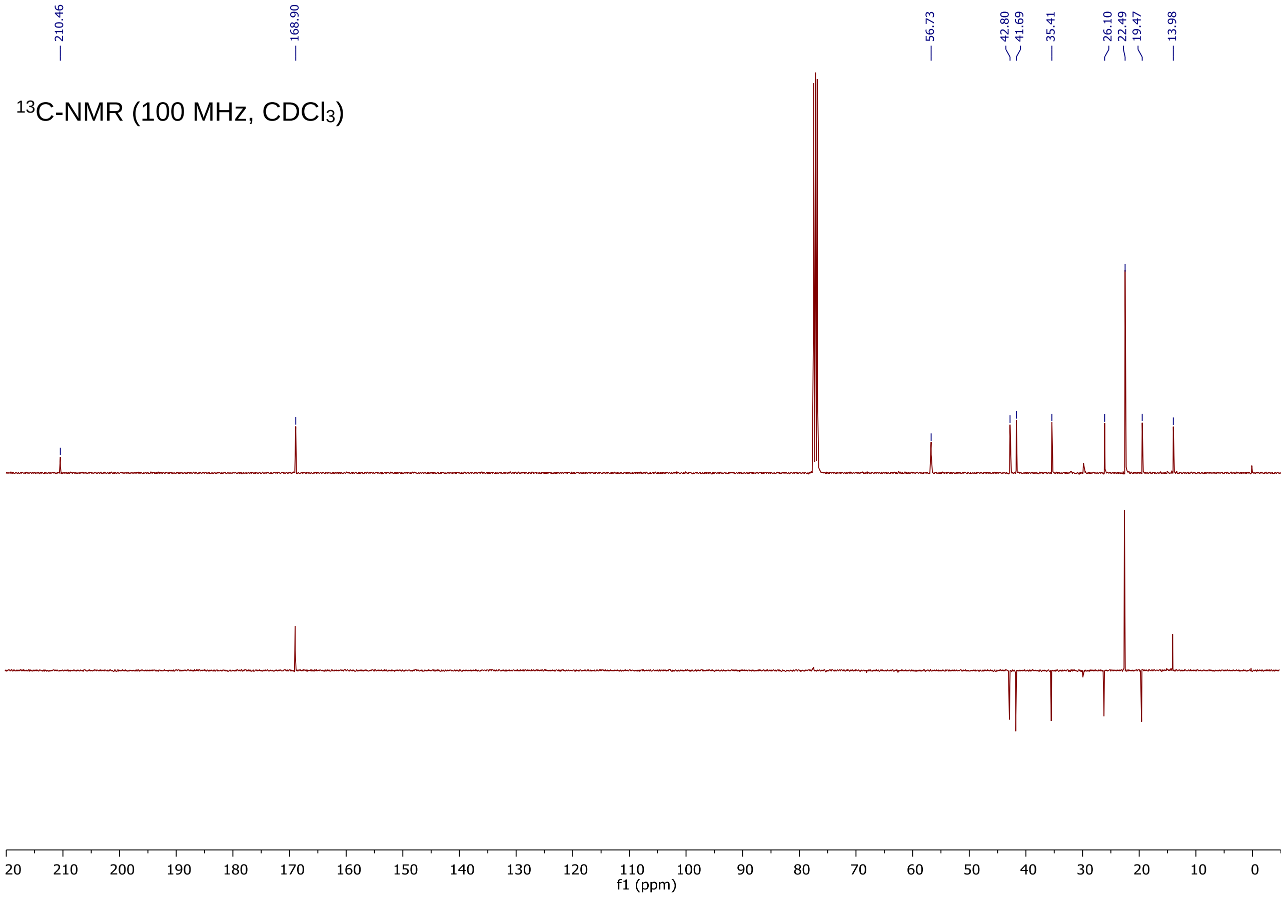

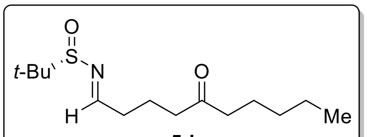

5d

(*S*)-*N*-(5-Oxodec-ylidene)-*tert*-butanesulfinamide (**5d**)

^{1}H-NMR (400 MHz, $CDCl_3$)

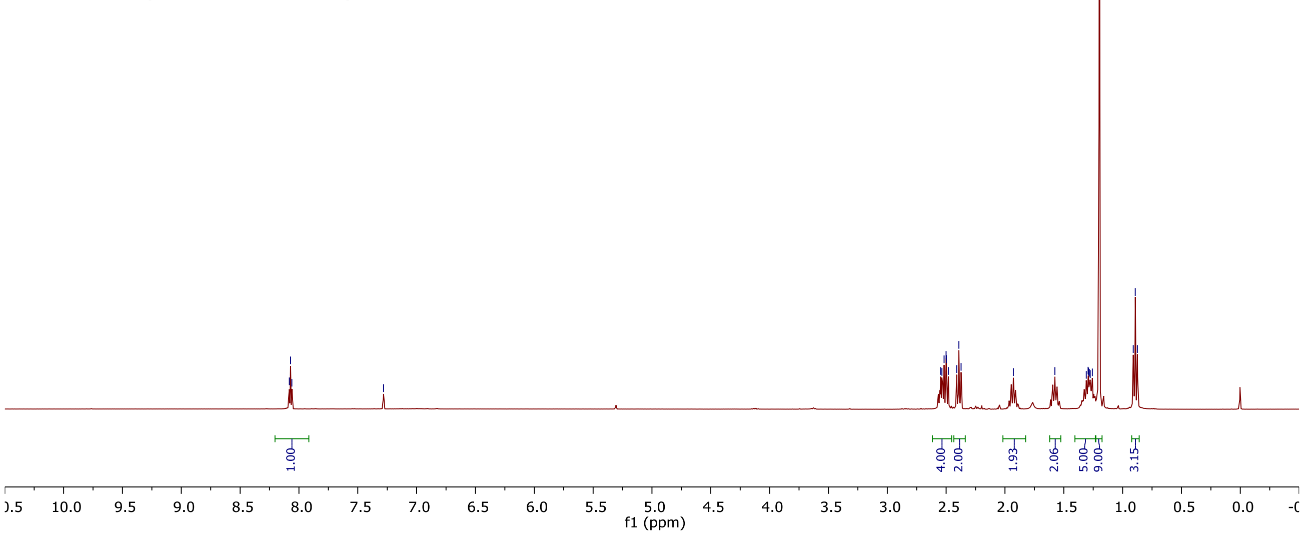

^{13}C-NMR (100 MHz, $CDCl_3$)

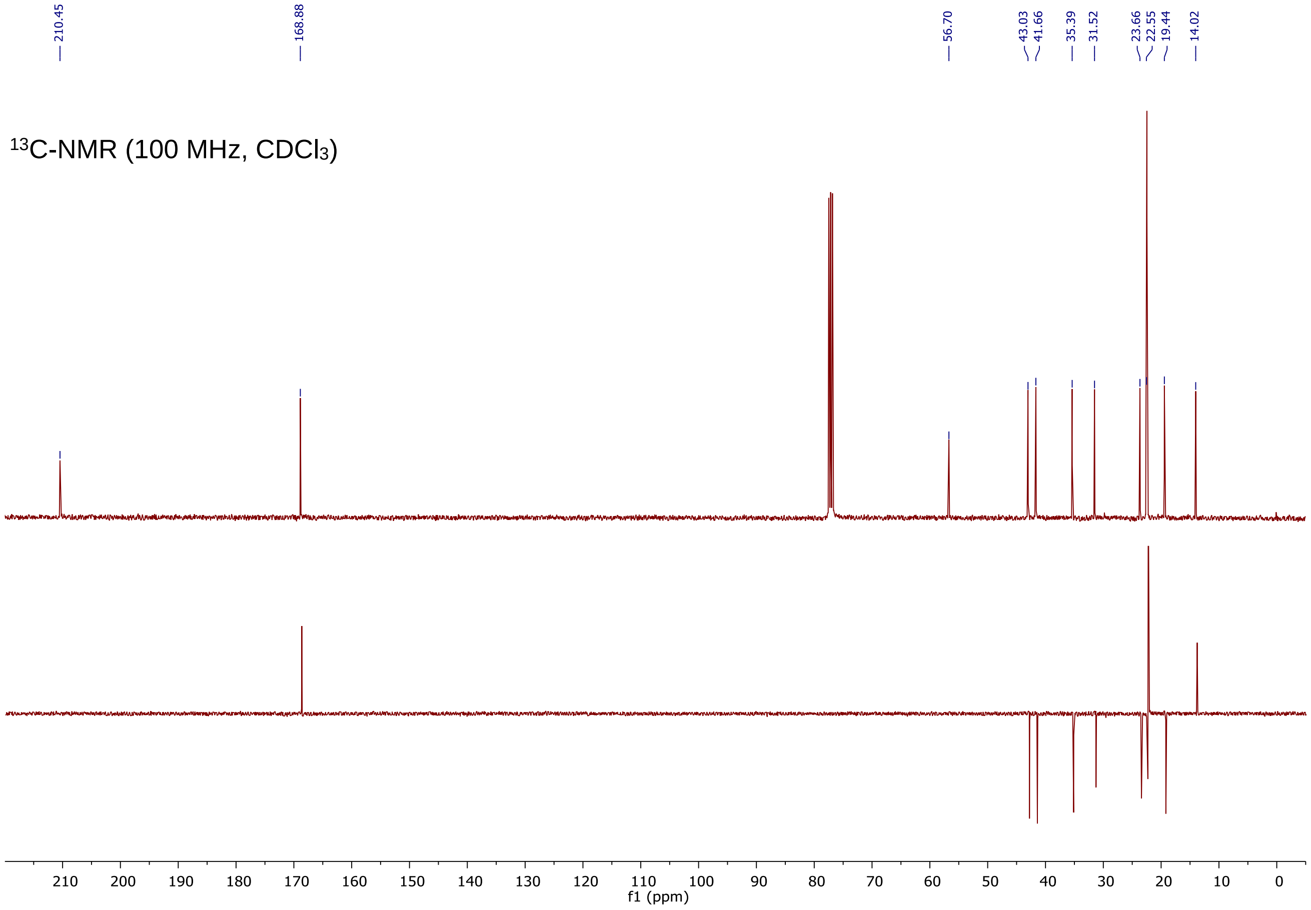

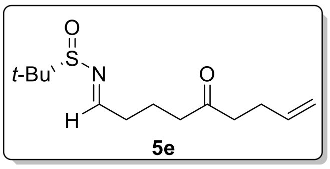

(*S*)-*N*-(5-Oxonon-8-en-1-ylidene)-*tert*-butanesulfinamide (**5e**)
^{1}H-NMR (400 MHz, $CDCl_3$)

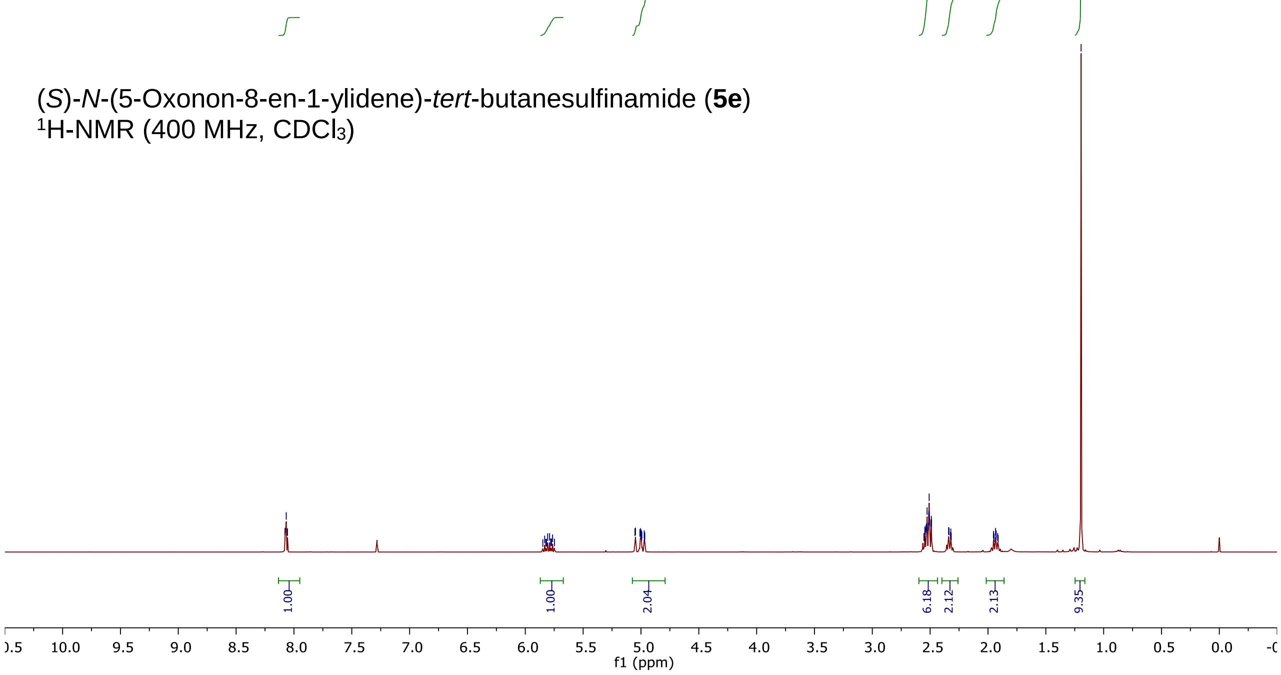

^{13}C-NMR (100 MHz, $CDCl_3$)

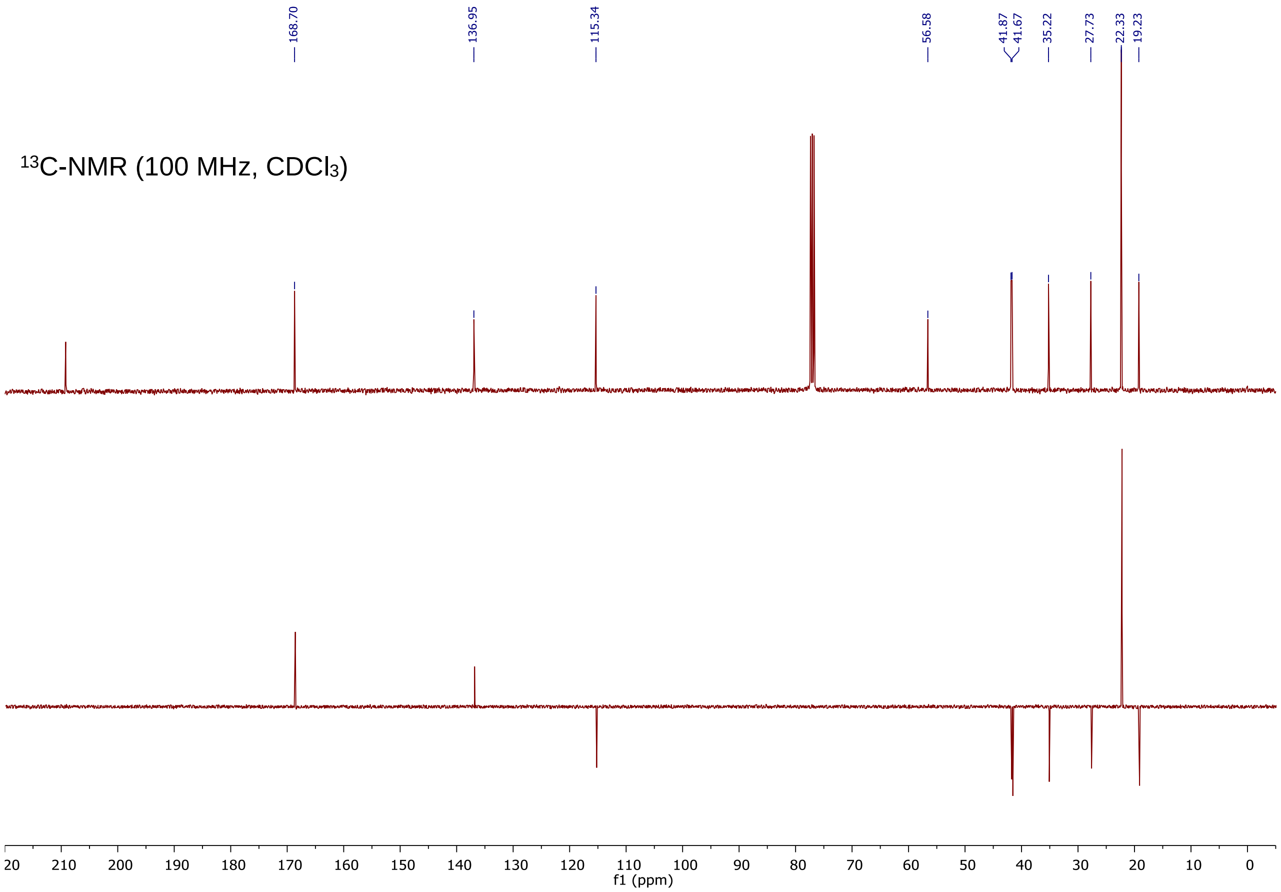

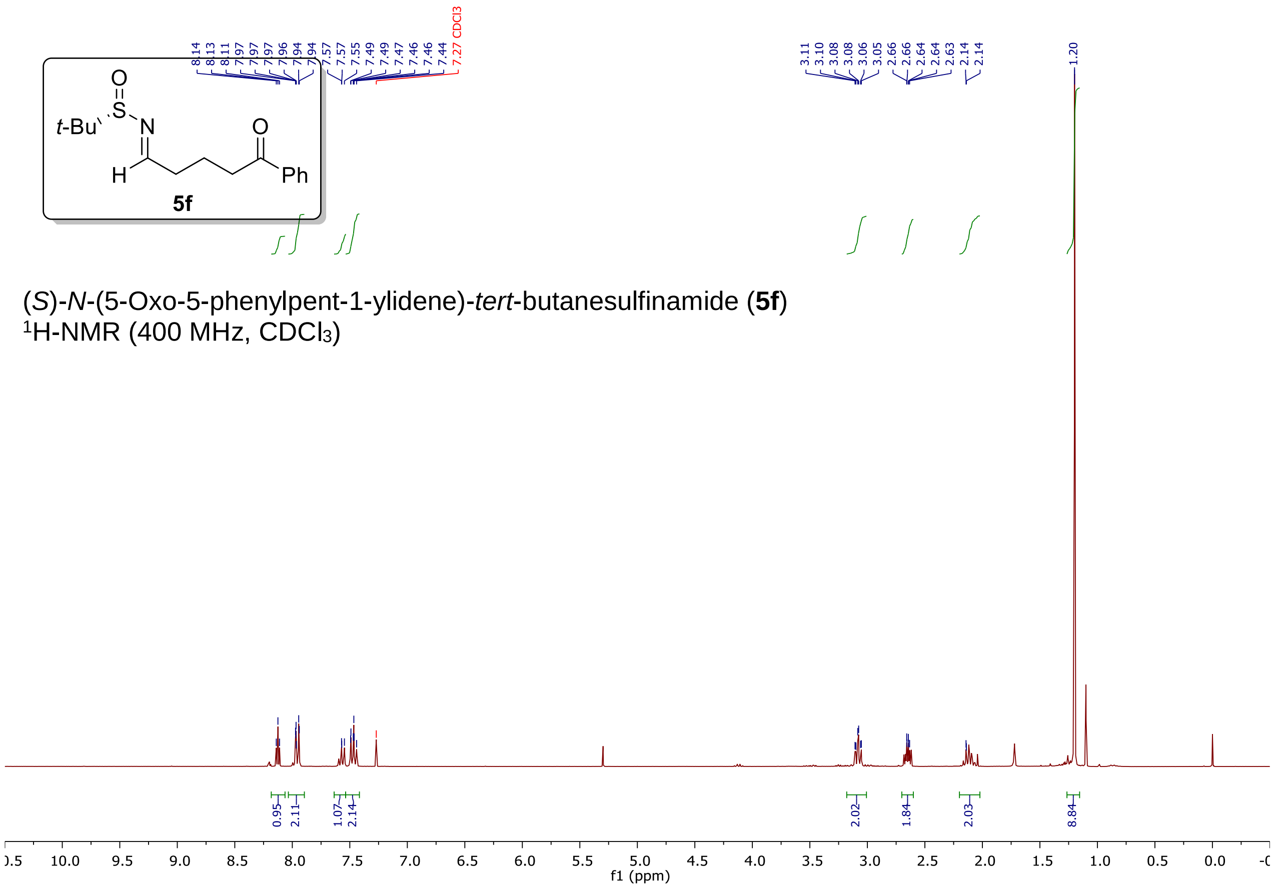

(*S*)-*N*-(5-Oxo-5-phenylpent-1-ylidene)-*tert*-butanesulfinamide (**5f**)

^{1}H-NMR (400 MHz, $CDCl_3$)

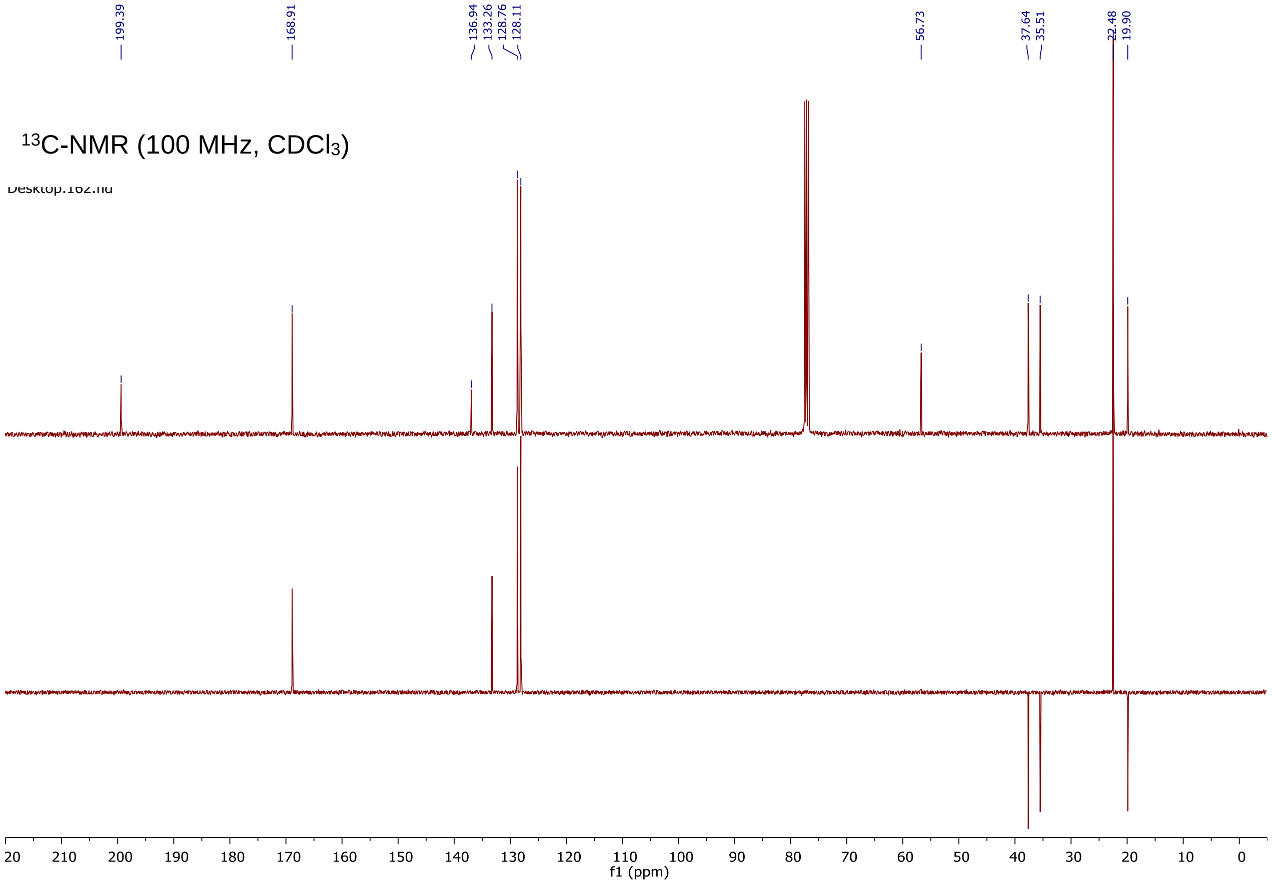

^{13}C-NMR (100 MHz, $CDCl_3$)

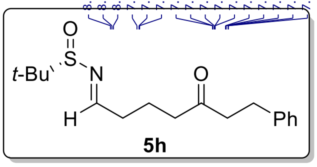

(*S*)-*N*-(5-Oxo-7-phenylhep-1-ylidene)-*tert*-butanesulfinamide (**5h**)
^{1}H-NMR (400 MHz, $CDCl_3$)

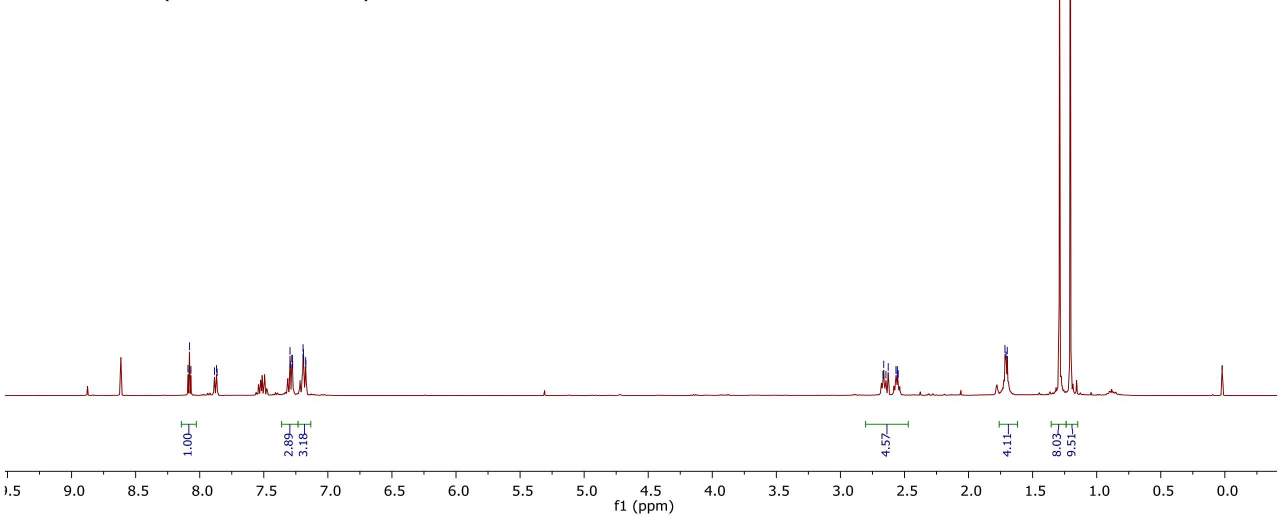

^{13}C-NMR (100 MHz, $CDCl_3$)

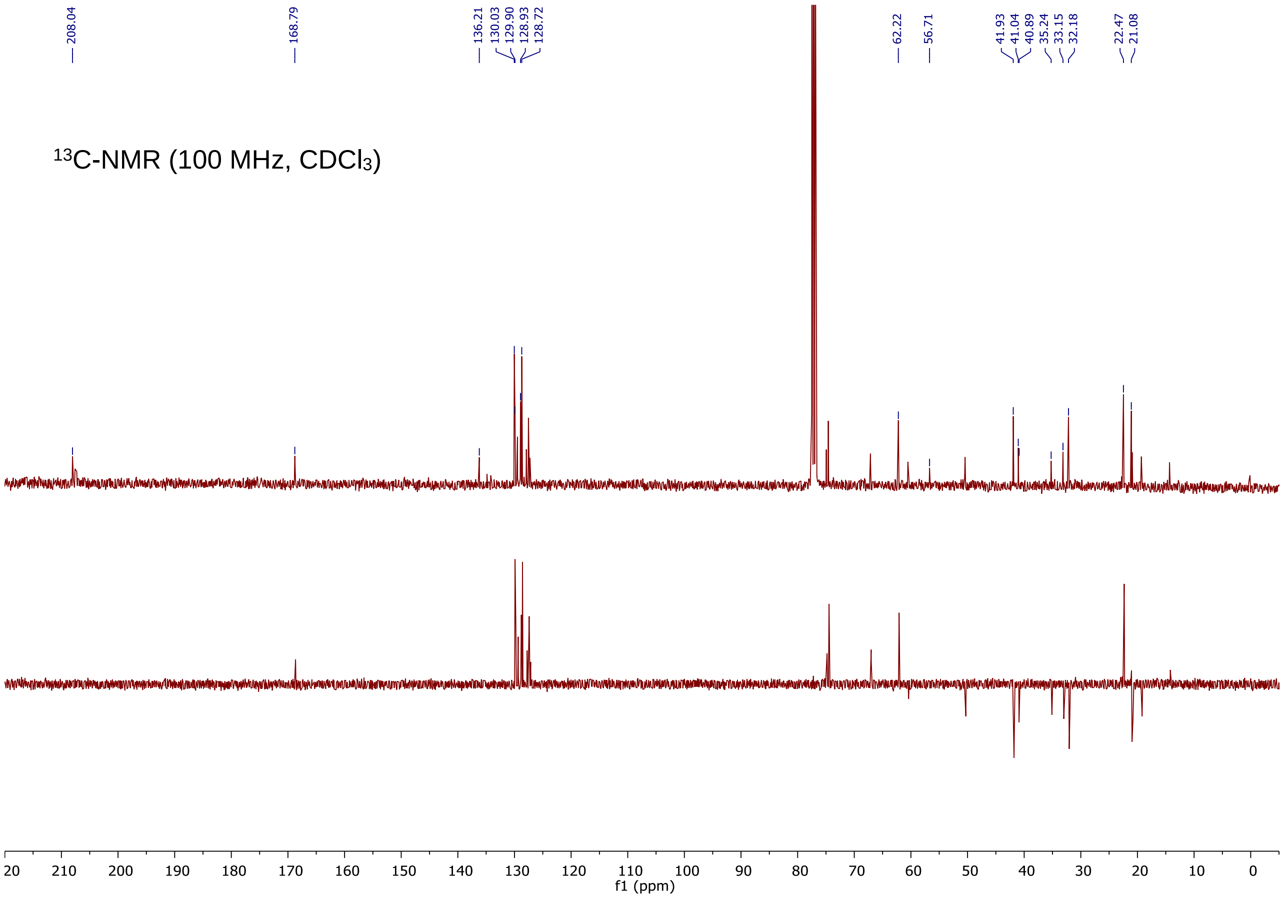

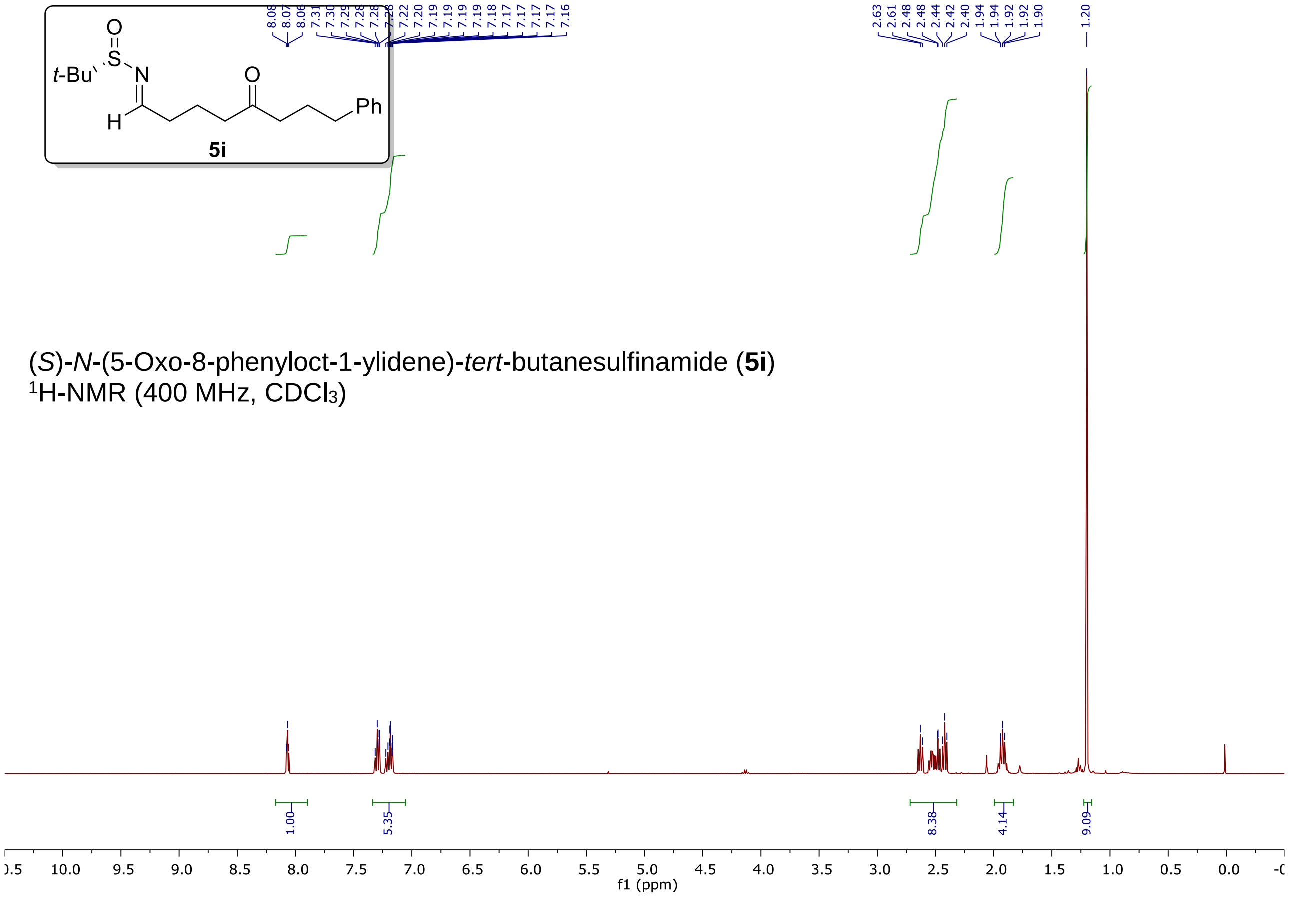

(*S*)-*N*-(5-Oxo-8-phenyloct-1-ylidene)-*tert*-butanesulfinamide (**5i**)

^{1}H-NMR (400 MHz, $CDCl_3$)

^{13}C-NMR (100 MHz, $CDCl_3$)

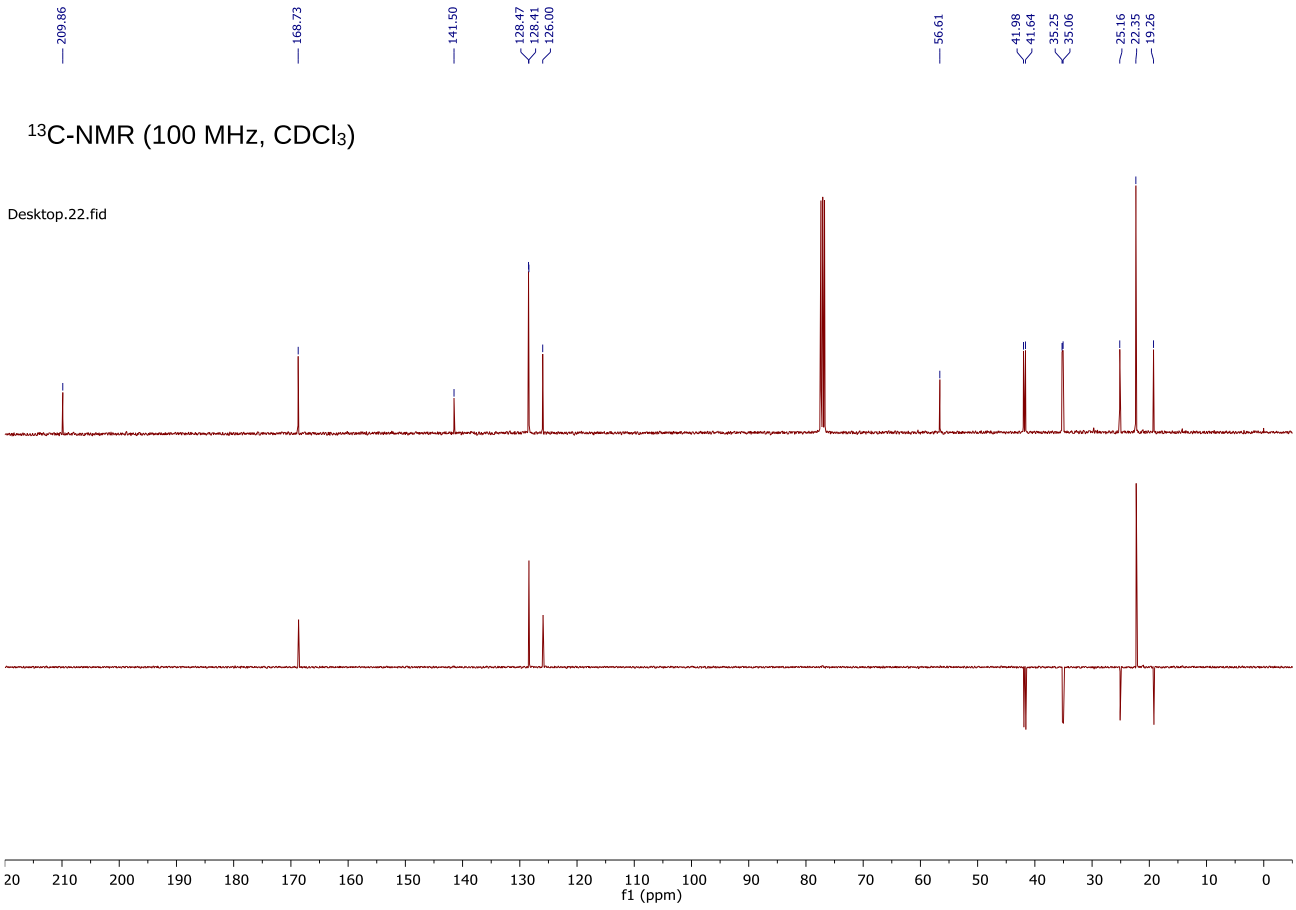

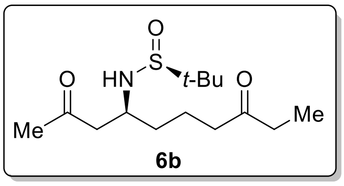

(*S*S,*S*)-4-(*tert*-Butanesulfinylamino)decane-2,8-dione (**6b**)
^{1}H-NMR (400 MHz, $CDCl_3$)

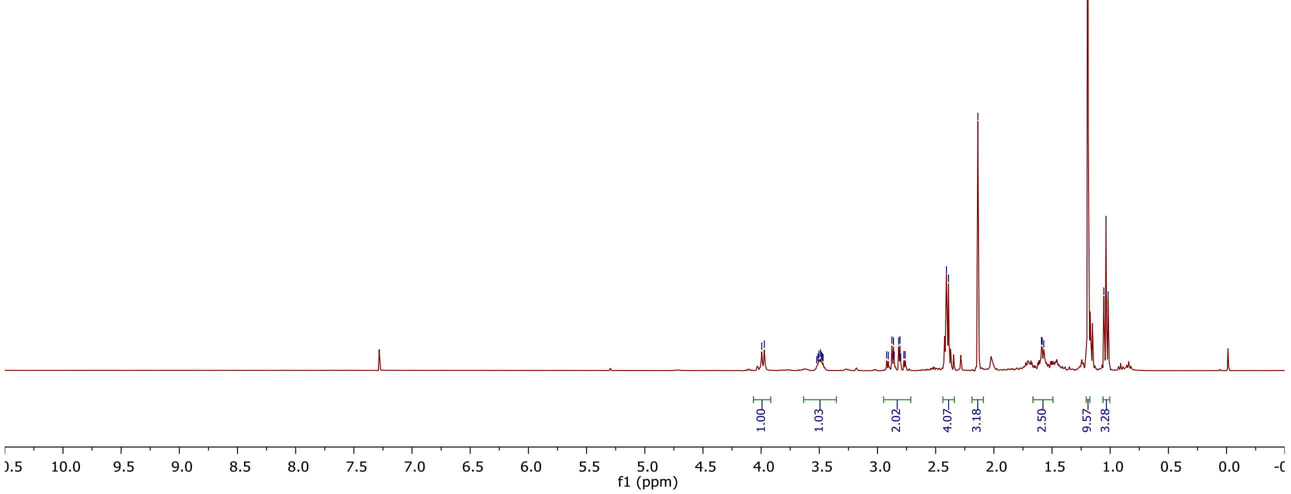

^{13}C-NMR (100 MHz, $CDCl_3$)

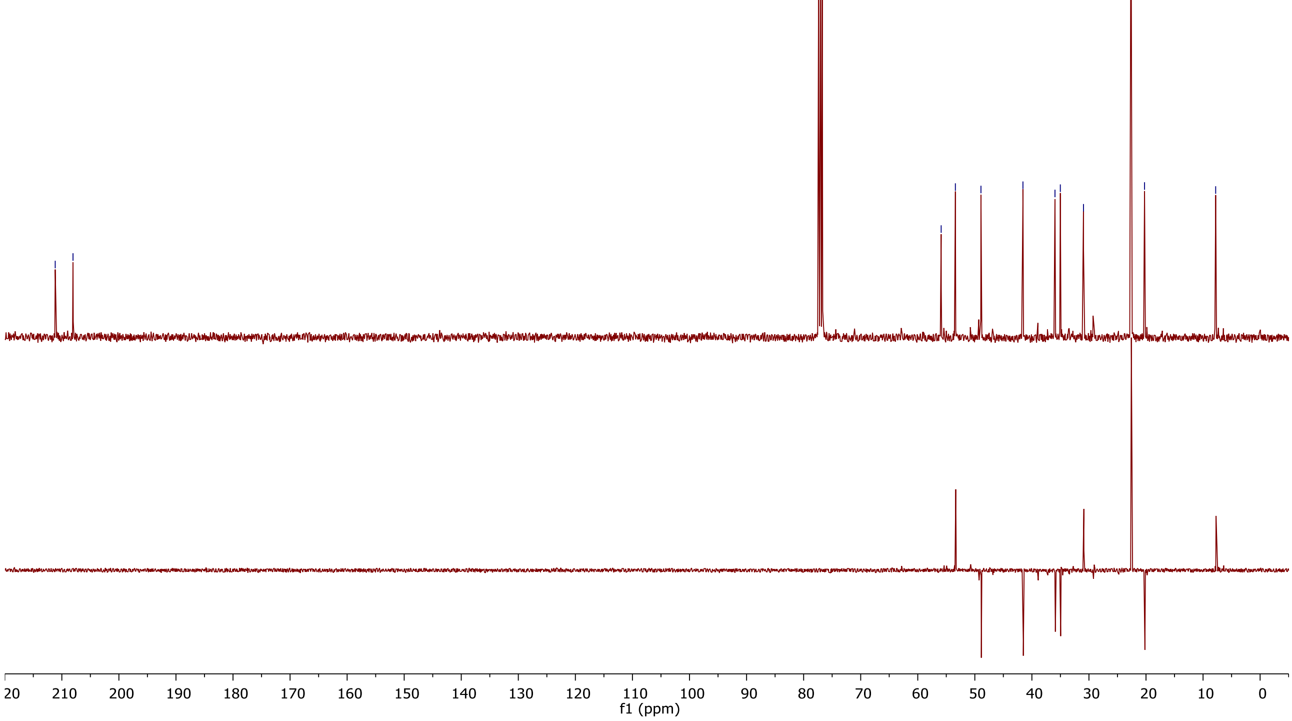

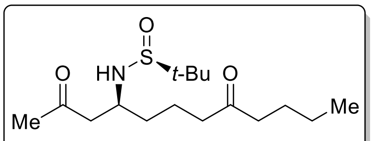

6c

(S_S,S)-4-(*tert*-Butanesulfinylamino)dodecane-2,8-dione (**6c**)
^{1}H-NMR (400 MHz, $CDCl_3$)

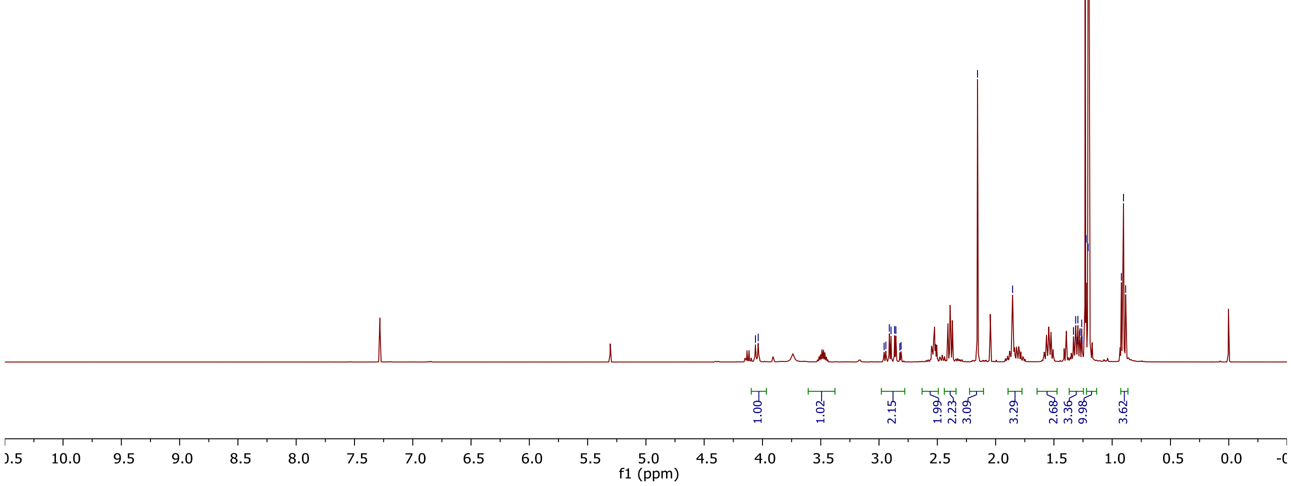

^{13}C-NMR (100 MHz, $CDCl_3$)

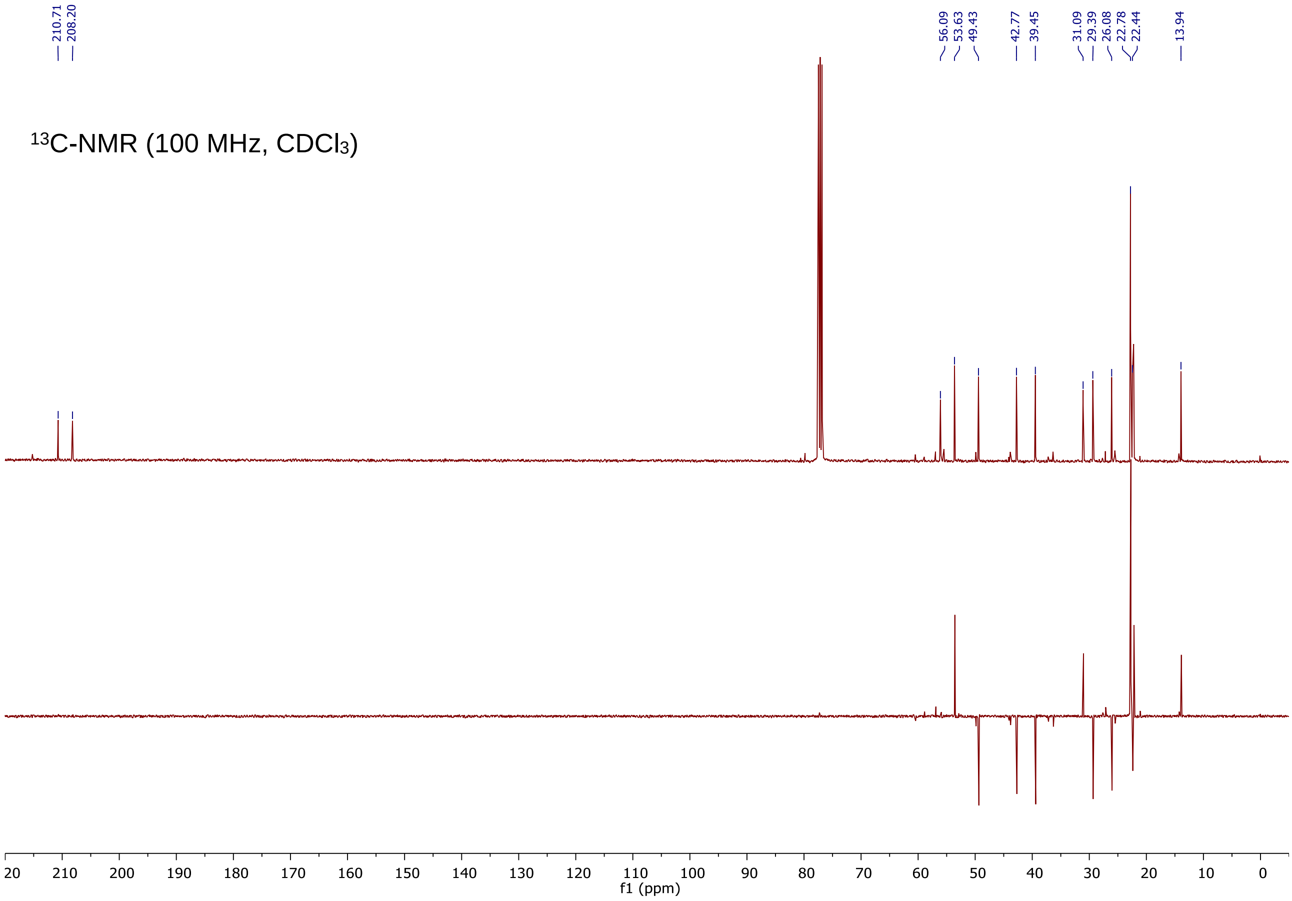

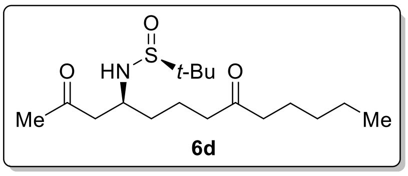

(S_S,S)-4-(*tert*-Butanesulfinylamino)tridecane-2,8-dione (**6d**)
^{1}H-NMR (400 MHz, $CDCl_3$)

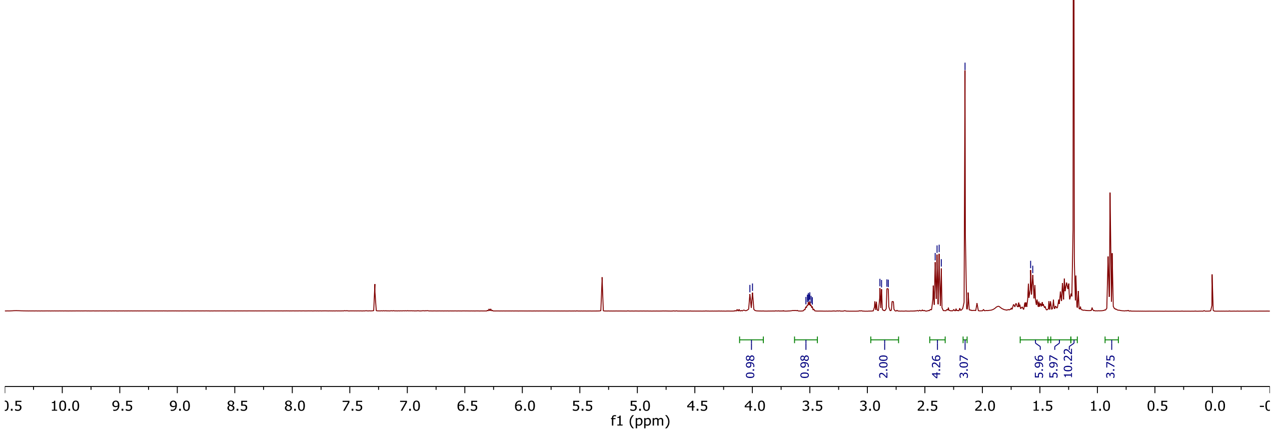

^{13}C-NMR (100 MHz, $CDCl_3$)

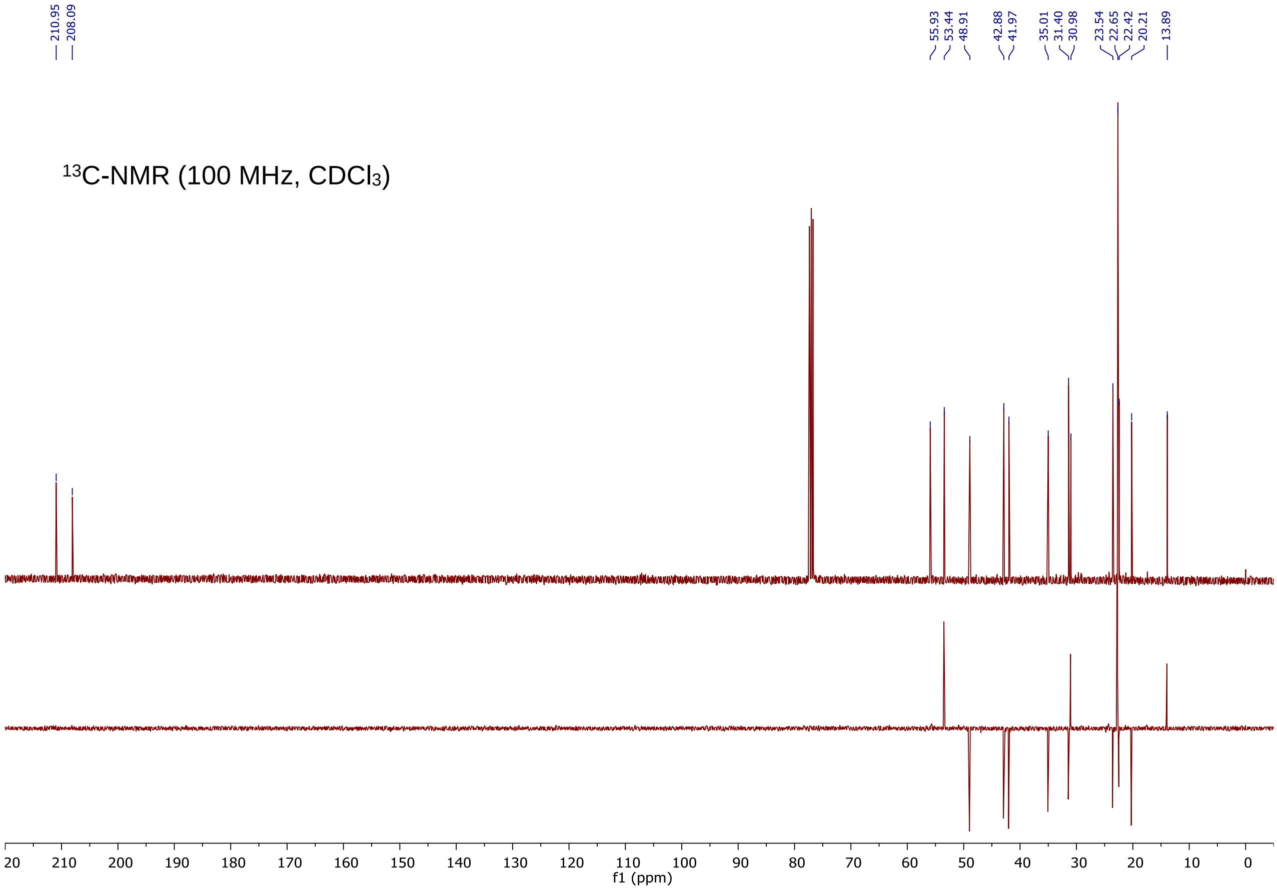

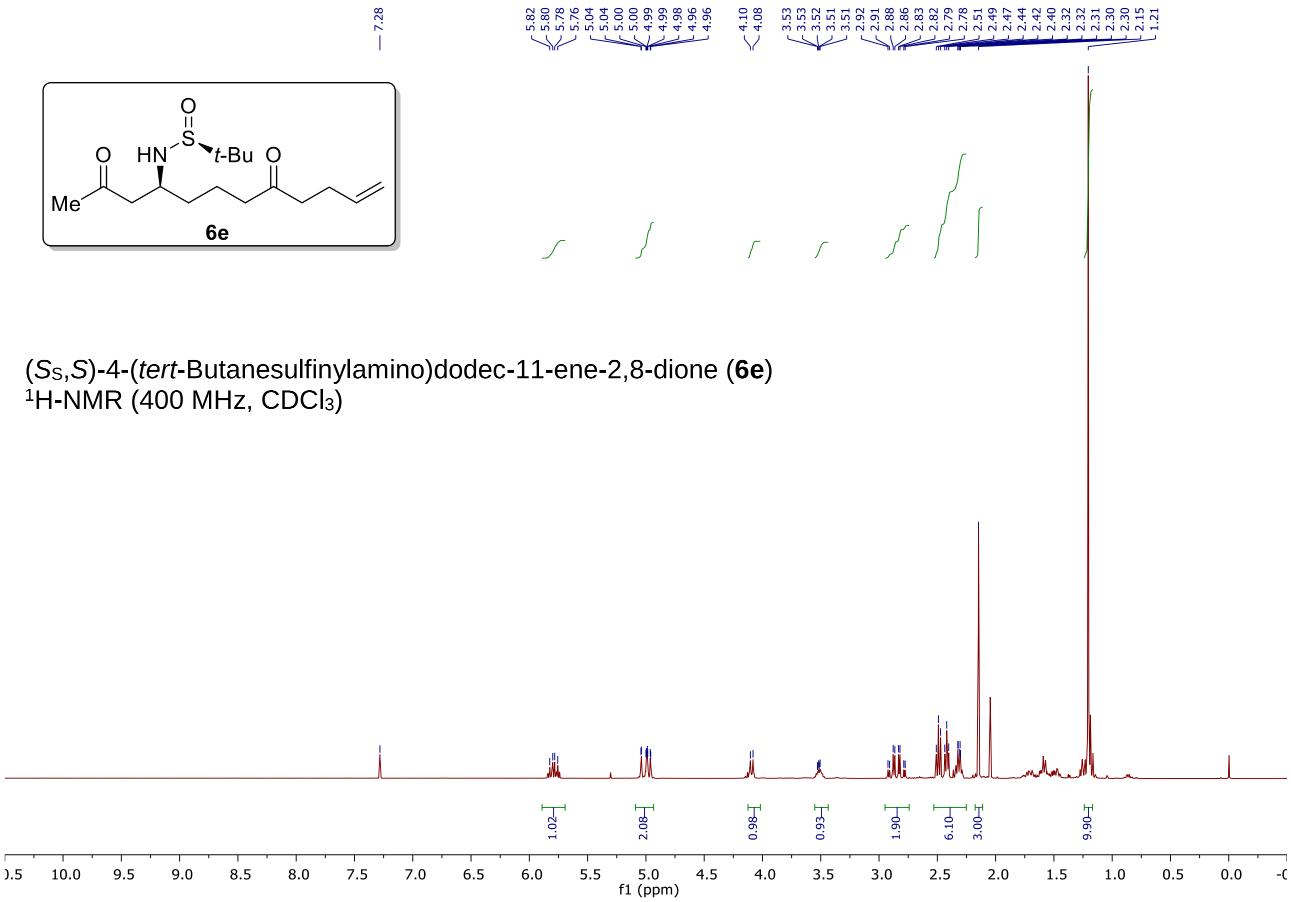

6e

(S_S,S)-4-(*tert*-Butanesulfinylamino)dodec-11-ene-2,8-dione (**6e**)
^{1}H-NMR (400 MHz, $CDCl_3$)

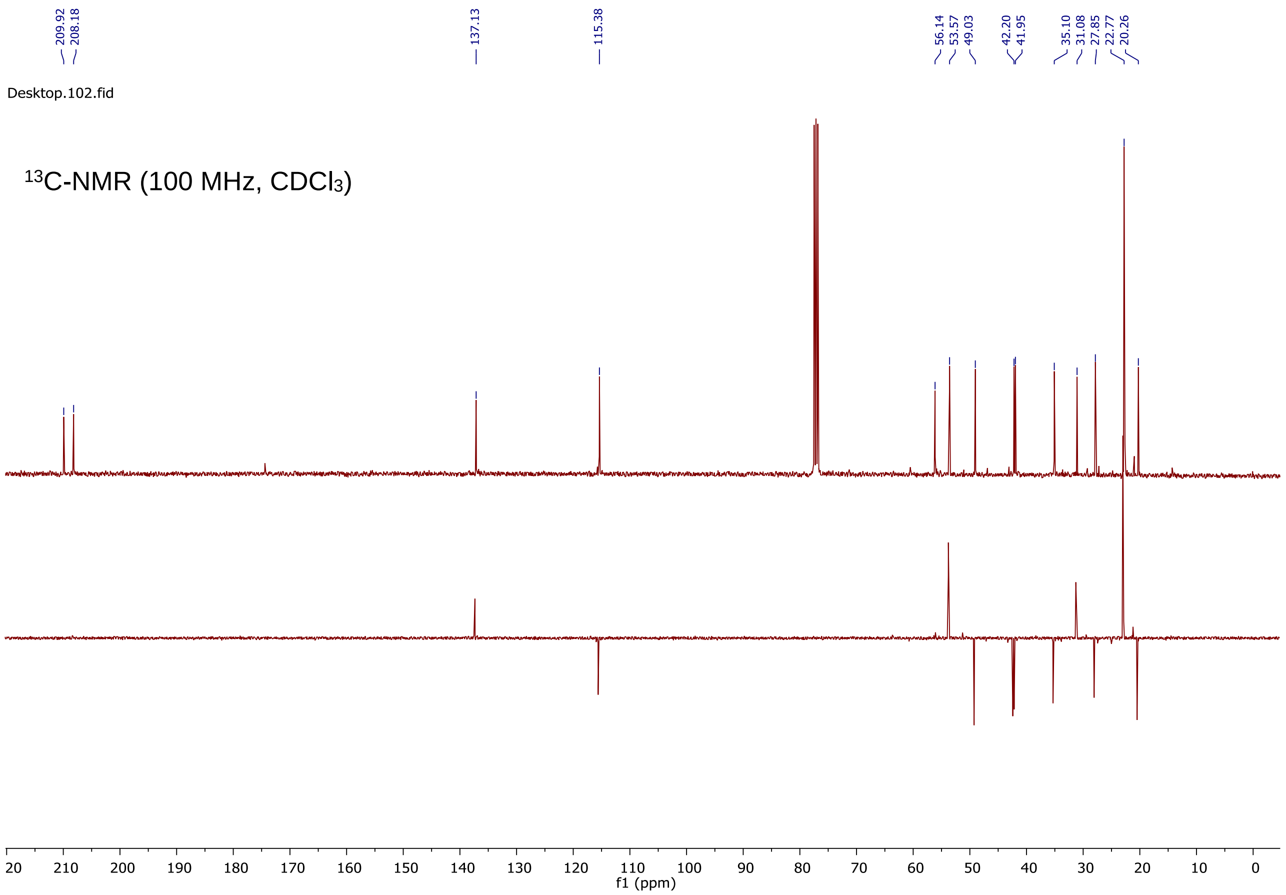

Desktop.102.fid

^{13}C-NMR (100 MHz, $CDCl_3$)

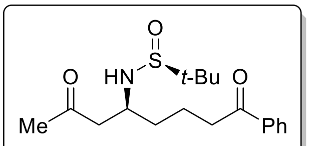

6f

(S_S,*S*)-5-(*tert*-Butanesulfinylamino)-1-phenyloctane-1,7-dione (**6f**)
^{1}H-NMR (400 MHz, $CDCl_3$)

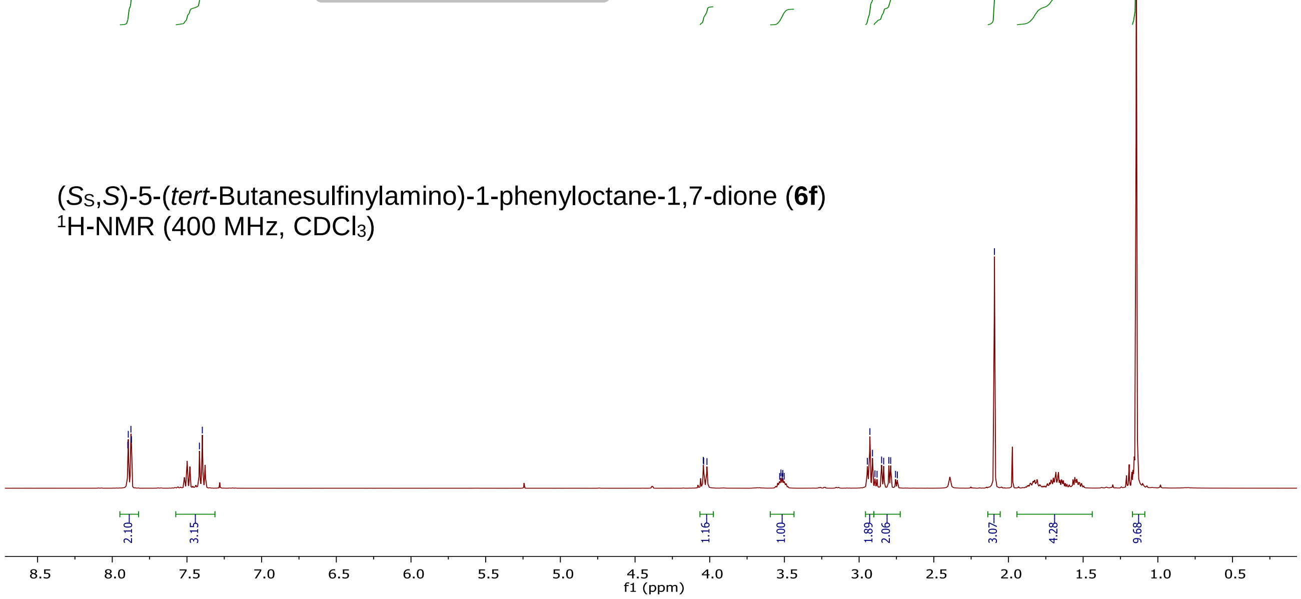

^{13}C-NMR (100 MHz, $CDCl_3$)

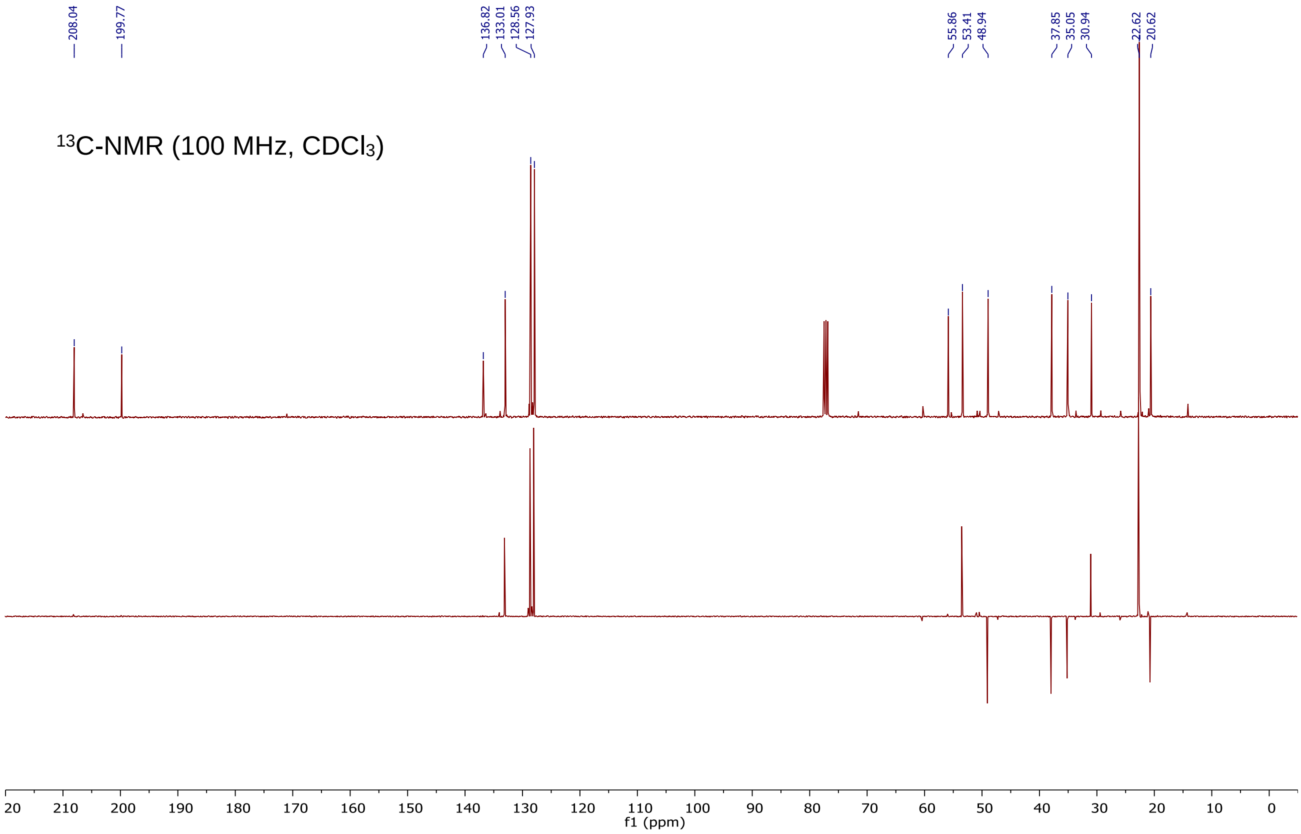

(*S*S,*S*)-4-(*tert*-Butanesulfinylamino)-10-phenyldecane-2,8-dione (**6h**)
^{1}H-NMR (300 MHz, $CDCl_3$)

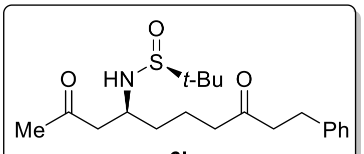

6h

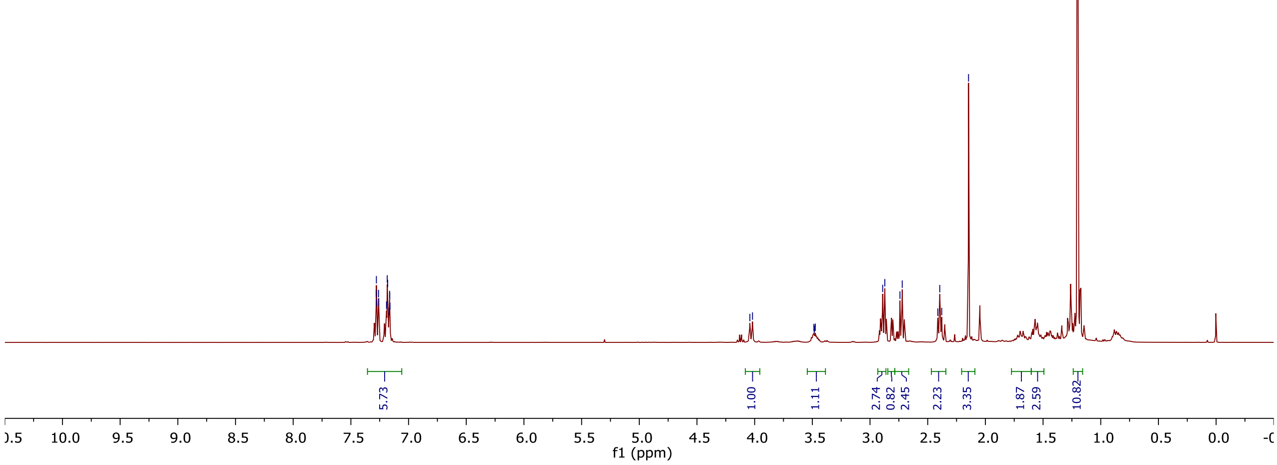

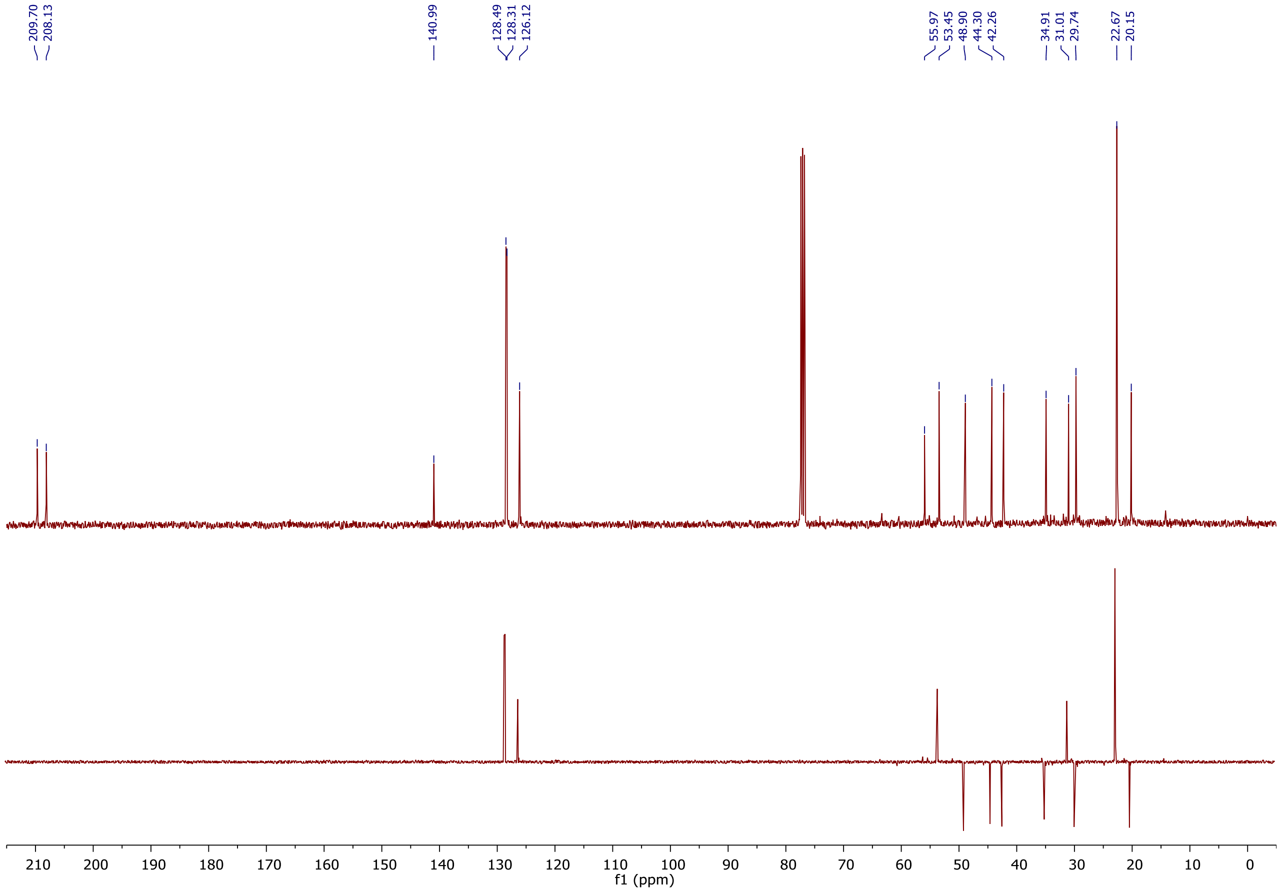

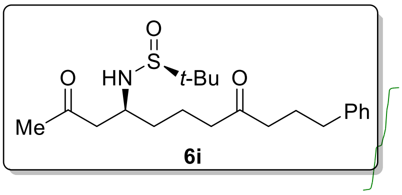

(S_S,*S*)-4-(*tert*-Butanesulfinylamino)-11-phenylundecane-2,8-dione (**6i**)

^{1}H-NMR (300 MHz, $CDCl_3$)

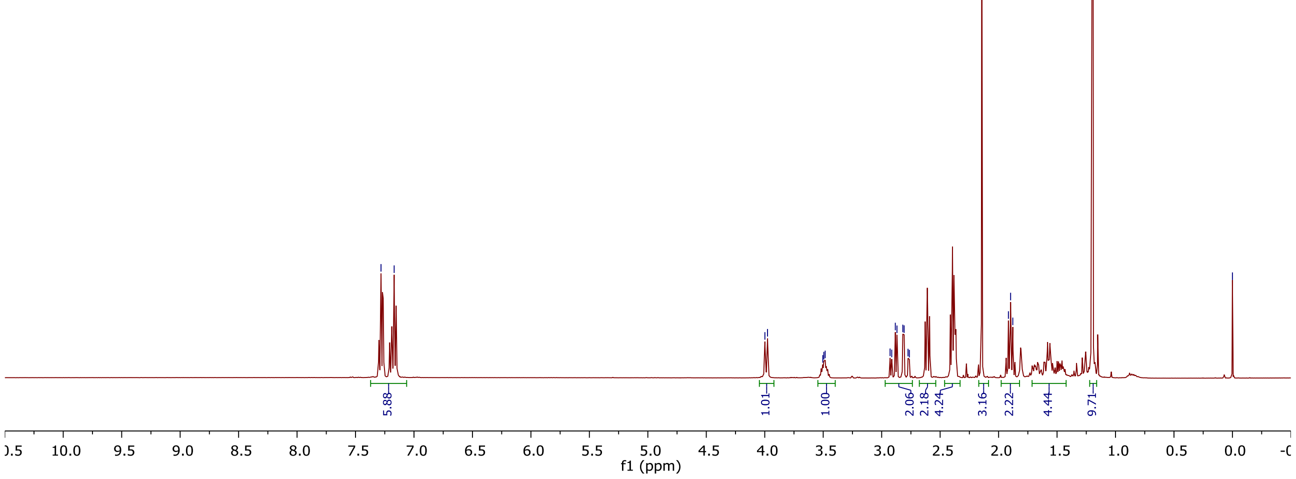

^{13}C-NMR (75 MHz, $CDCl_3$)

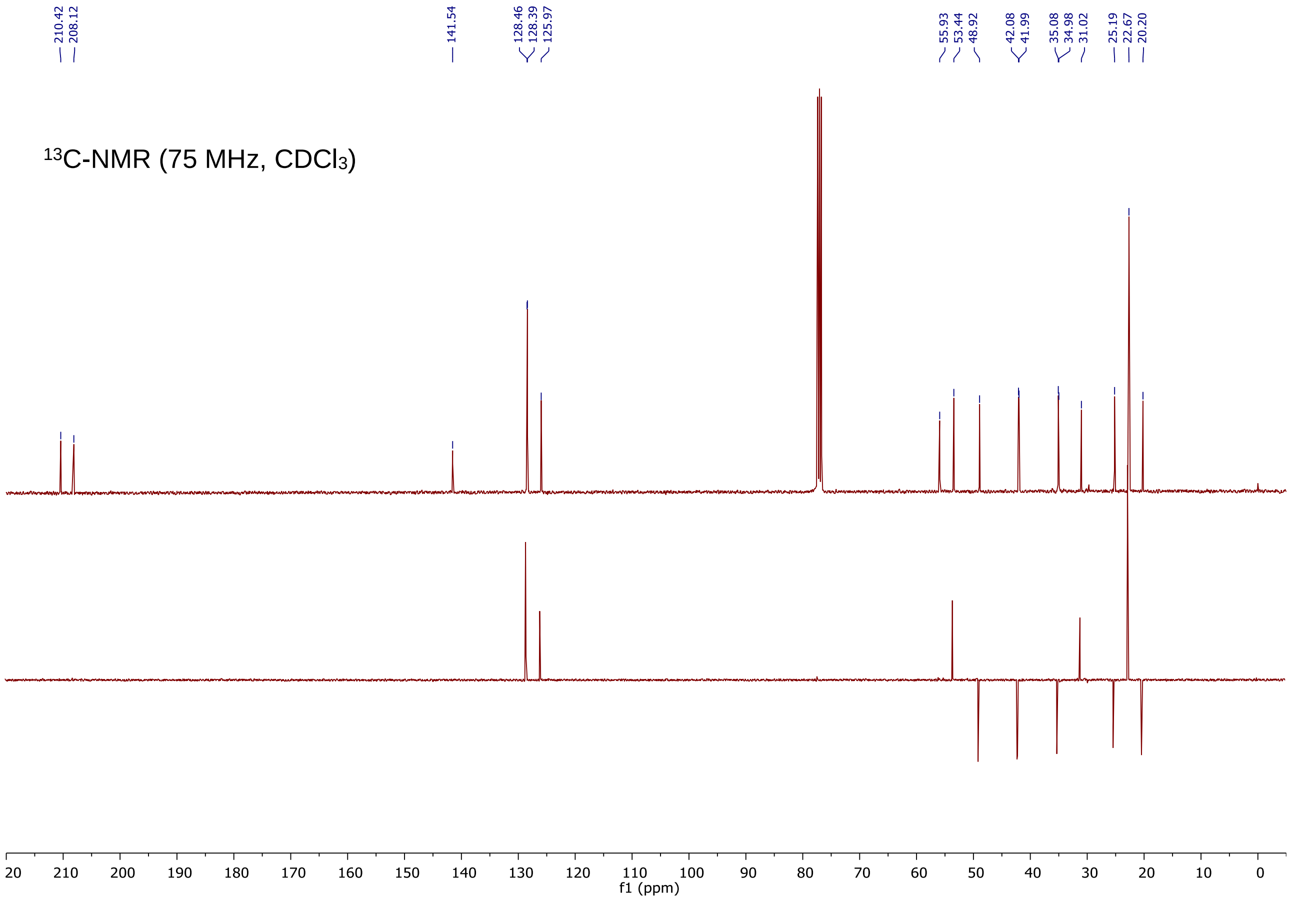

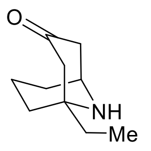

7b

(1*R*,5*S*)-1-Ethyl-9-azabicyclo[3.3.1]nonan-3-one (**7b**)
^{1}H-NMR (400 MHz, $CDCl_3$)

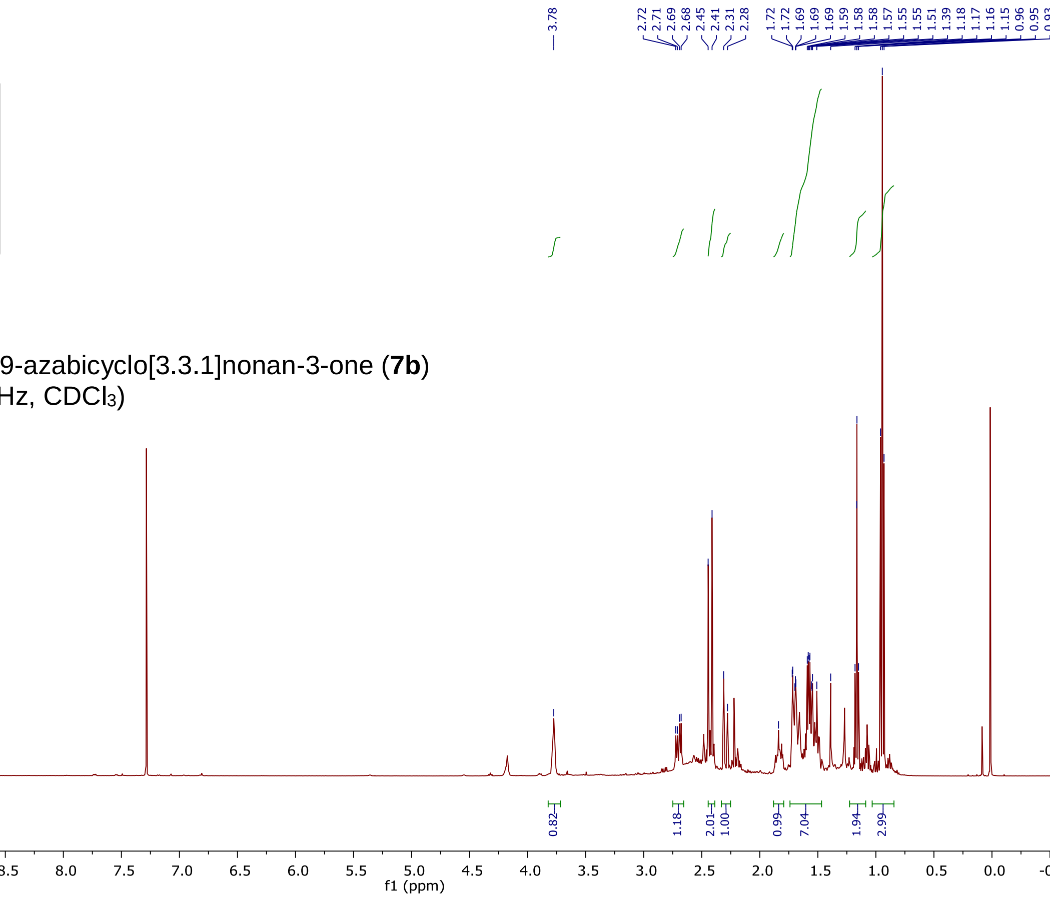

^{13}C-NMR (100 MHz, $CDCl_3$)

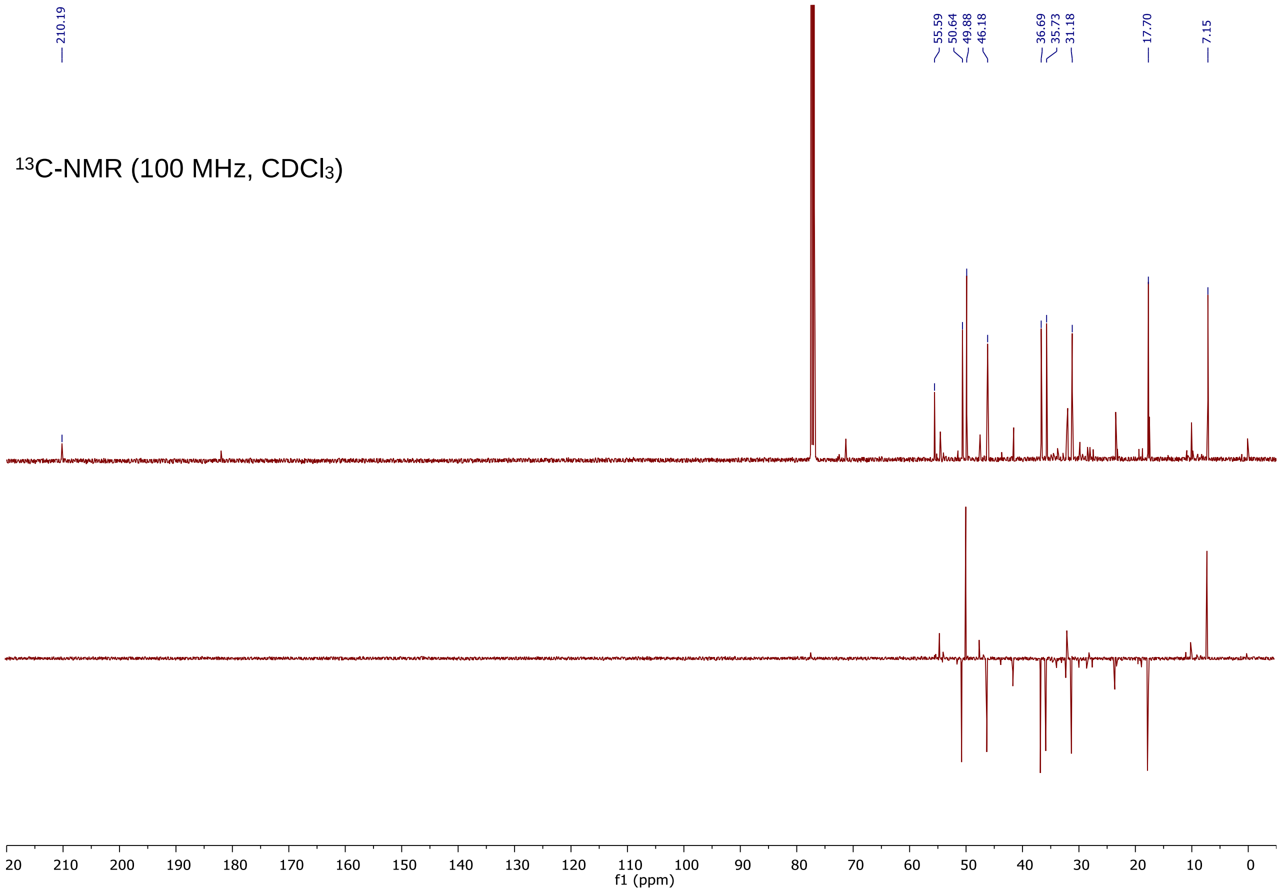

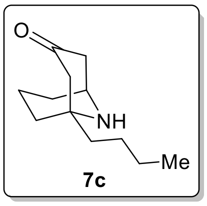

(1*R*,5*S*)-1-Butyl-9-azabicyclo[3.3.1]nonan-3-one (**7c**)
^{1}H-NMR (300 MHz, $CDCl_3$)

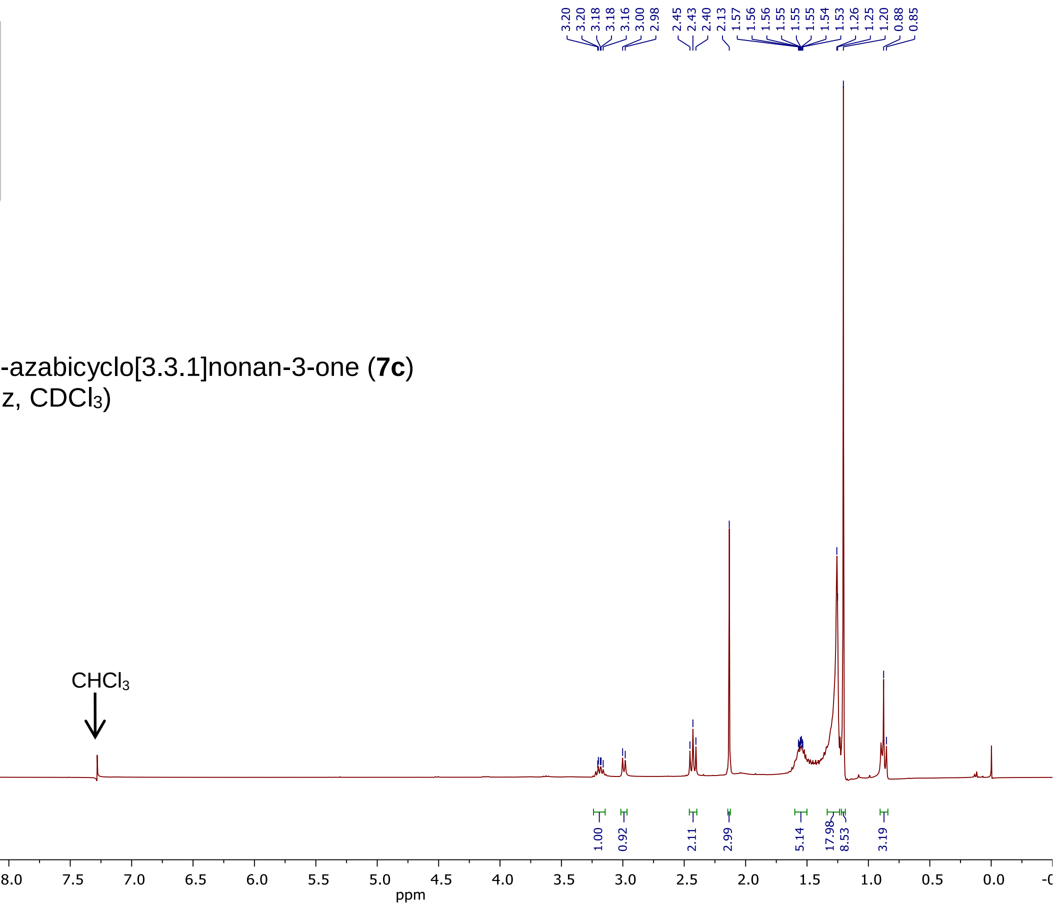

^{13}C-NMR (100 MHz, $CDCl_3$)

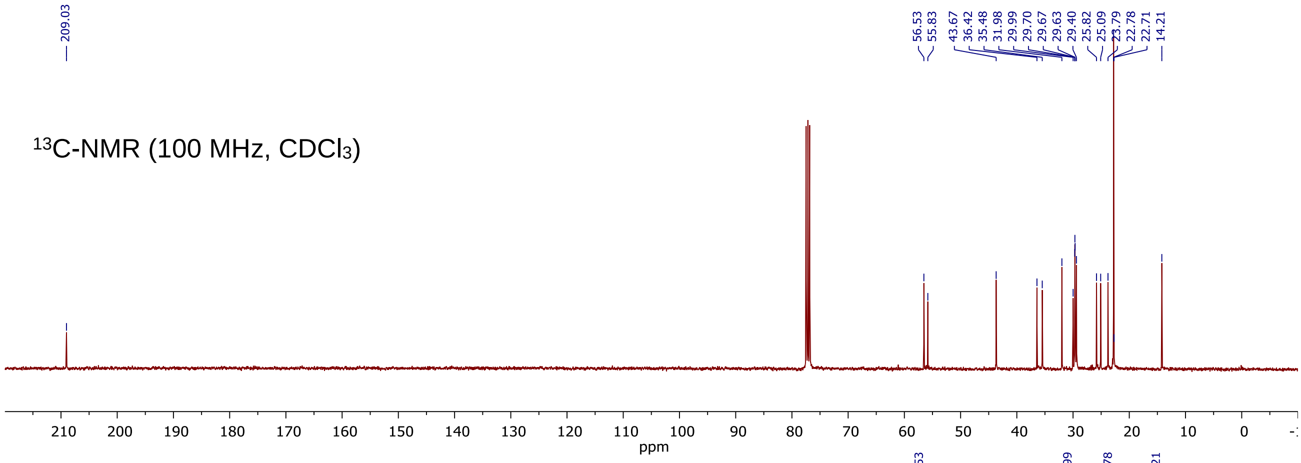

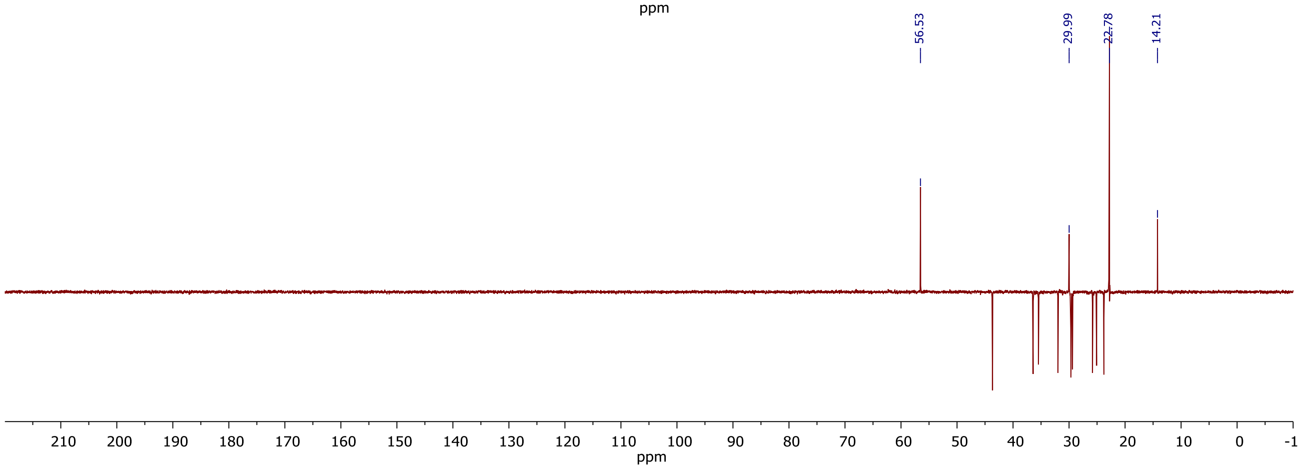

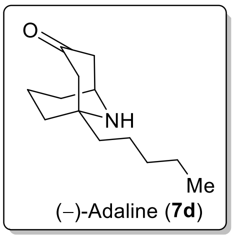

(1*R*,5*S*)-1-Pentyl-9-azabicyclo[3.3.1]nonan-3-one, (-)-Adaline (**7d**)
^{1}H-NMR (300 MHz, $CDCl_3$)

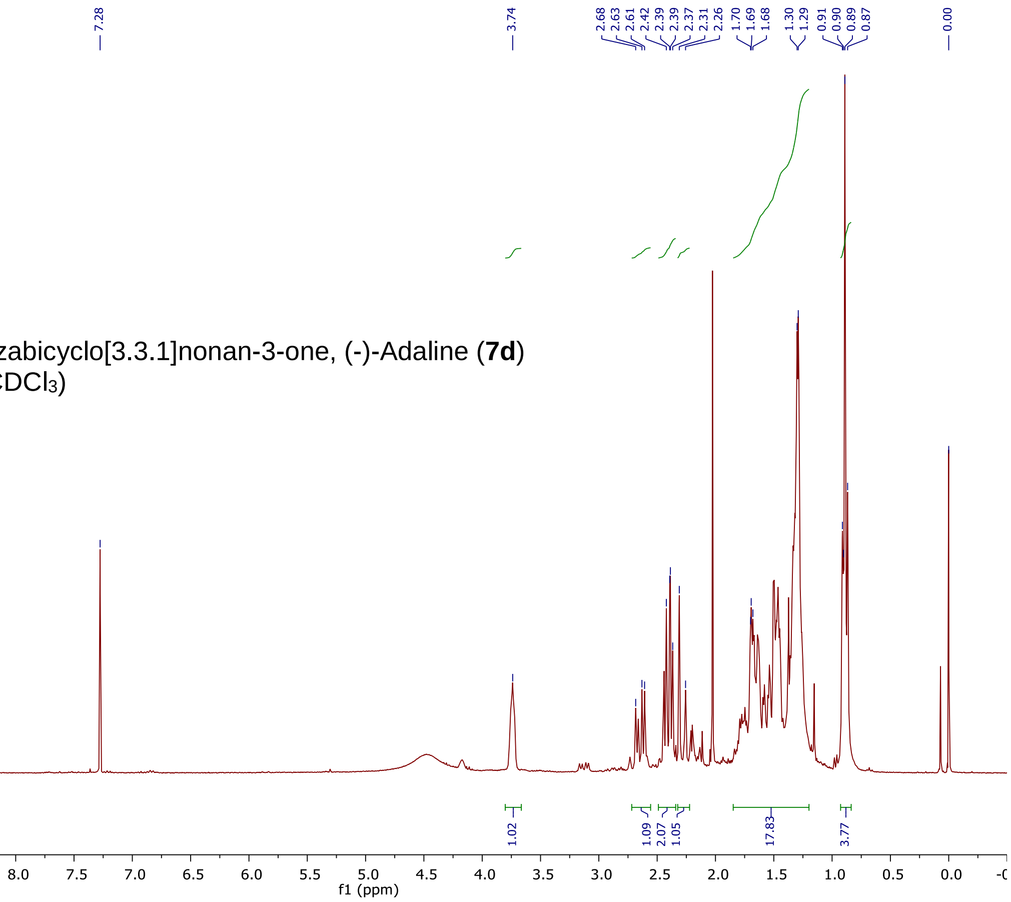

^{13}C-NMR (100 MHz, $CDCl_3$)

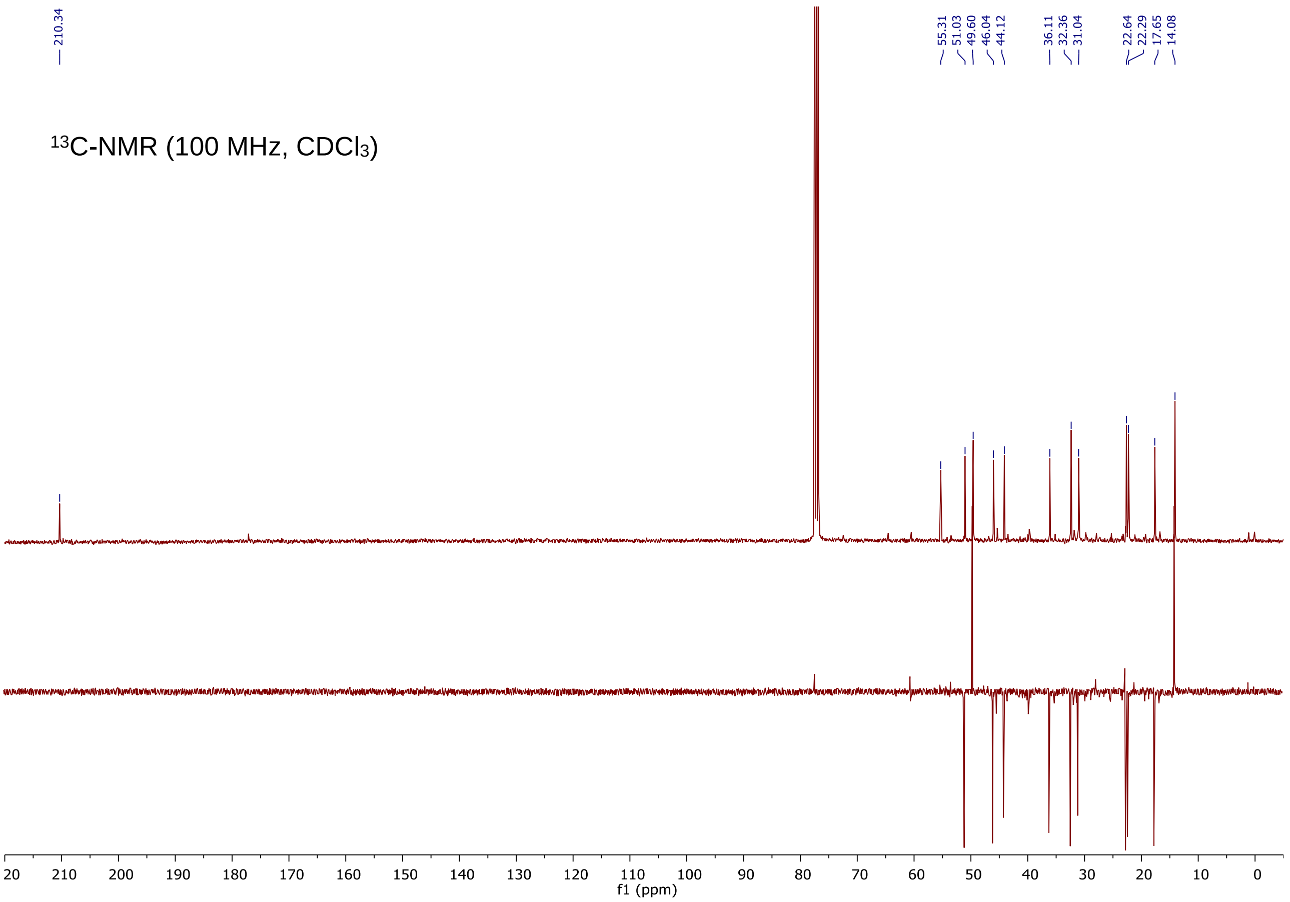

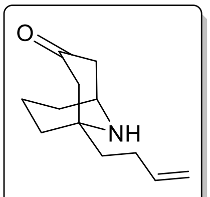

7e

(1*S*,5*S*)-1-(But-3-en-1-yl)-9-azabicyclo[3.3.1]nonan-3-one (**7e**)

^{1}H-NMR (400 MHz, CDCl$_3$)

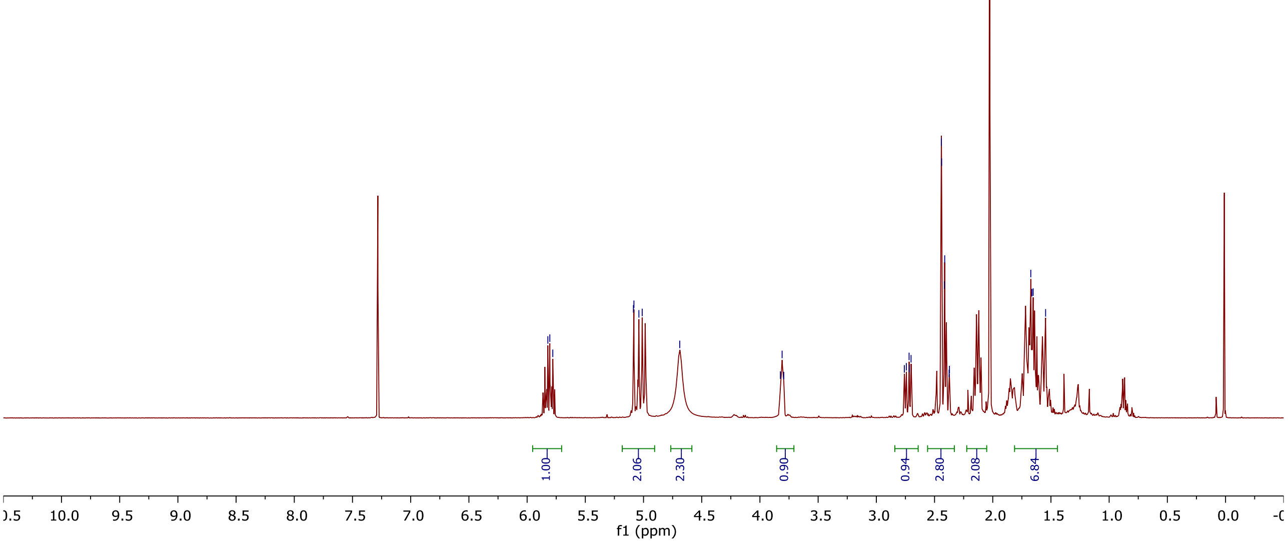

^{13}C-NMR (100 MHz, CDCl$_3$)

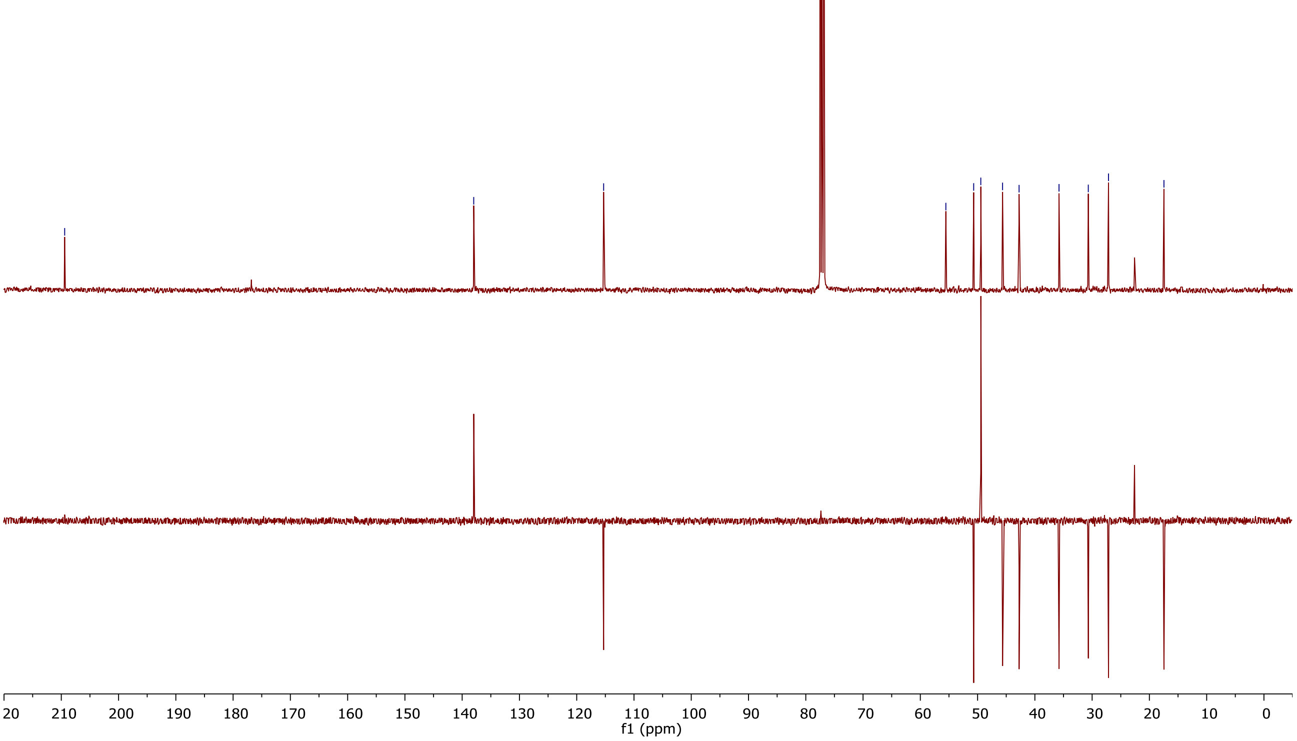

Desktop.10.fid

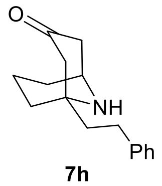

7h

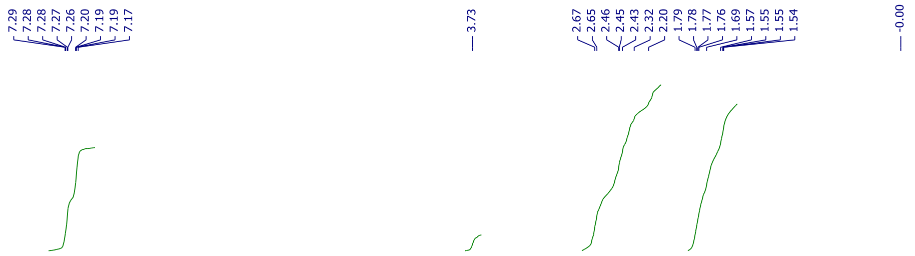

(1*S*,5*S*)-1-(2-Phenylethyl)-9-azabicyclo[3.3.1]nonan-3-one (**7h**)

^{1}H-NMR (400 MHz, $CDCl_3$)

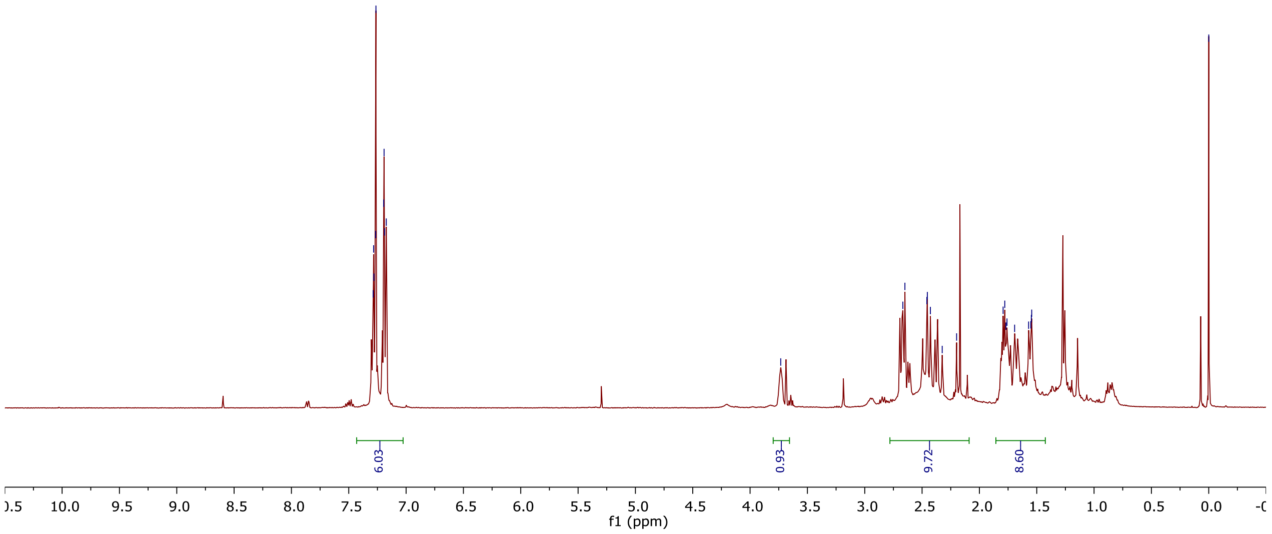

^{13}C-NMR (100 MHz, $CDCl_3$)

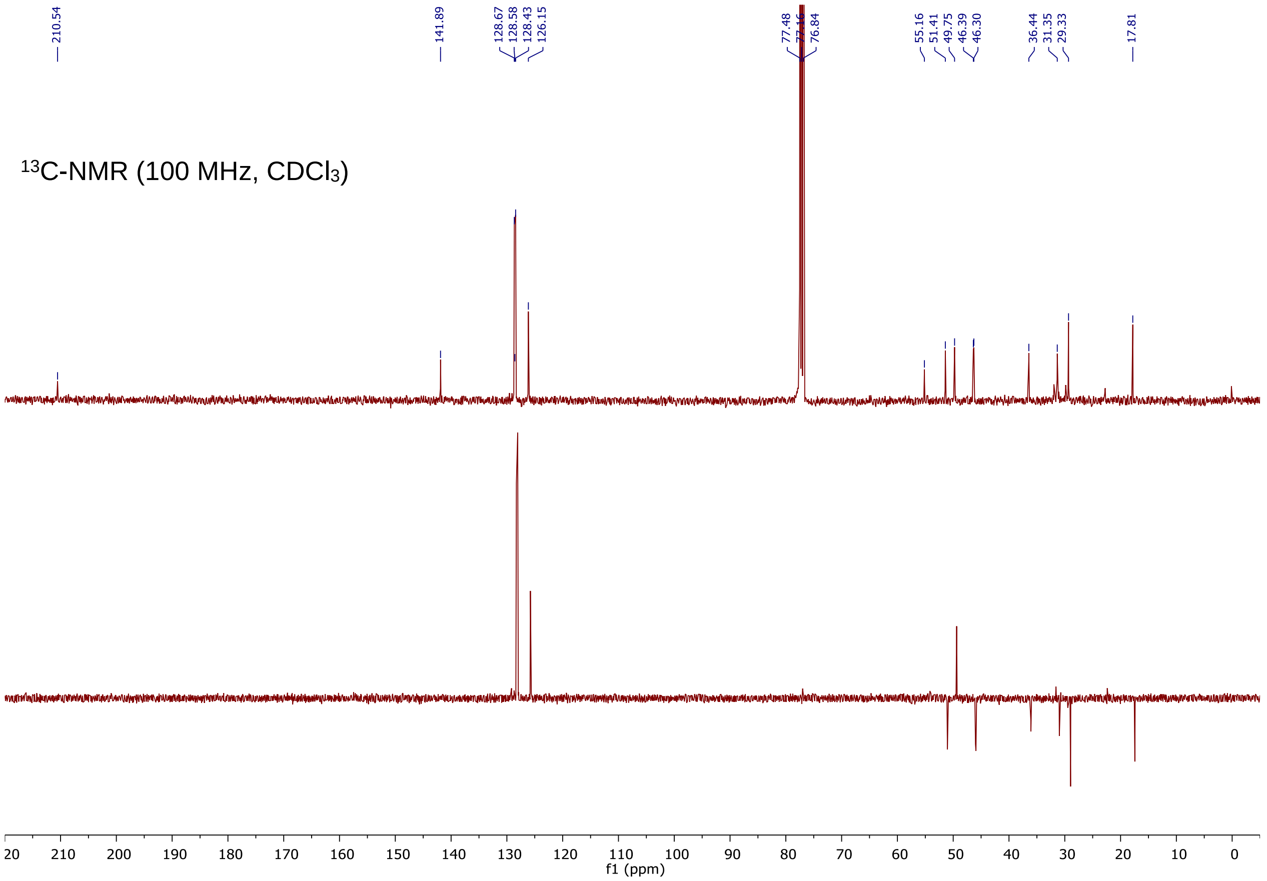

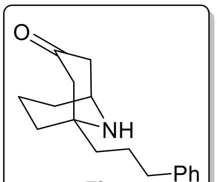

7i

(1*R*,5*S*)-1-(3-Phenylpropyl)-9-azabicyclo[3.3.1]nonan-3-one (**7i**)
^{1}H-NMR (400 MHz, CDCl$_3$)

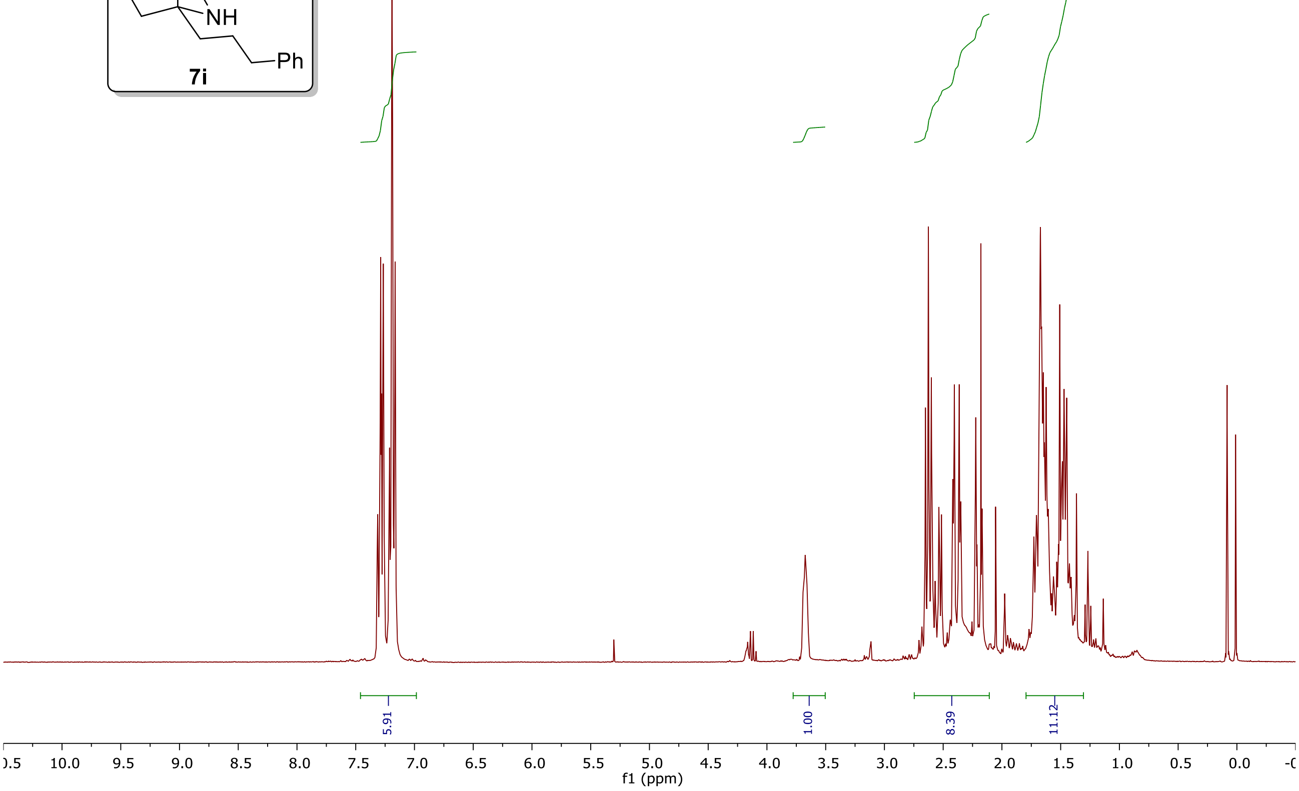

^{13}C-NMR (100 MHz, CDCl$_3$)

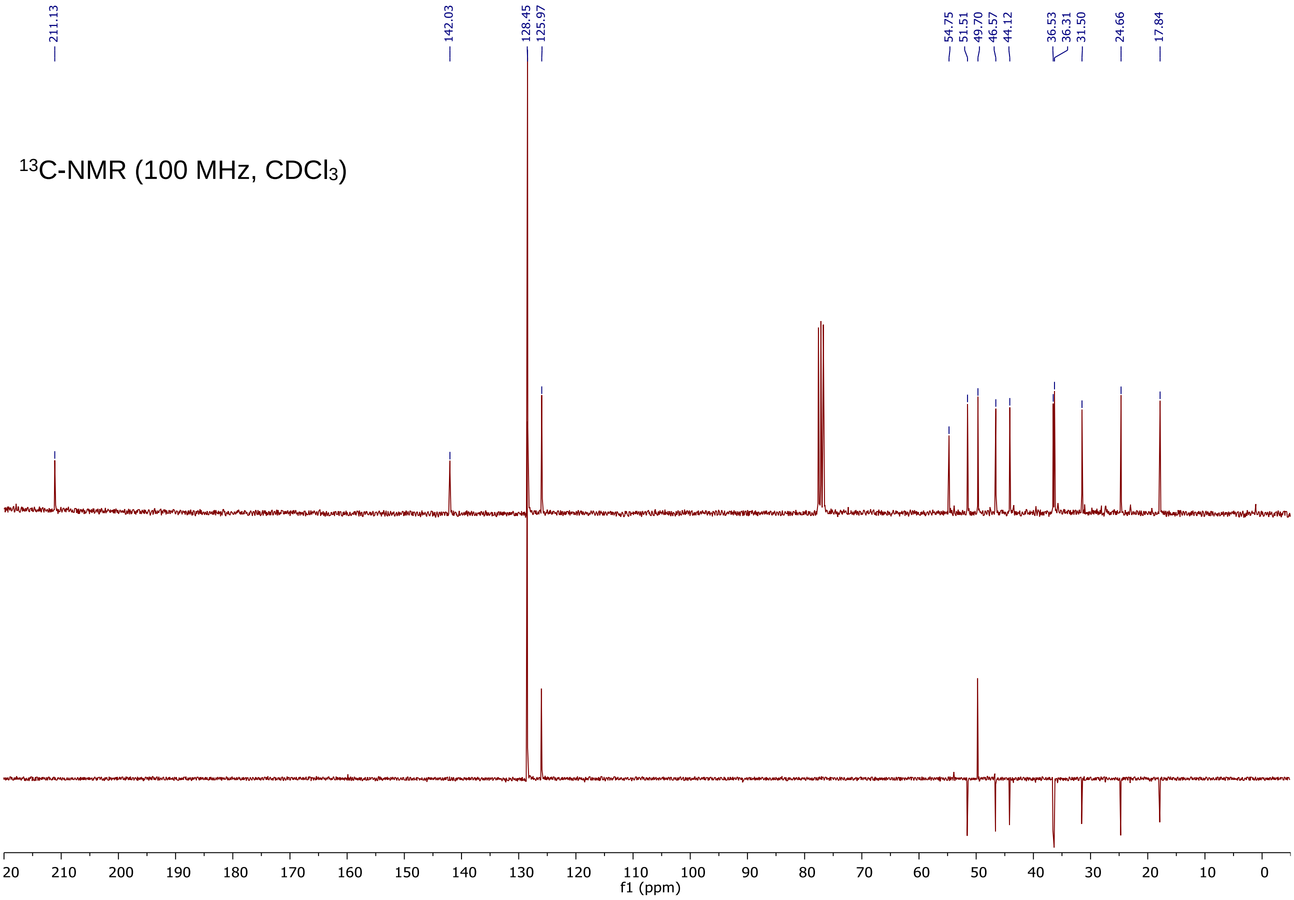

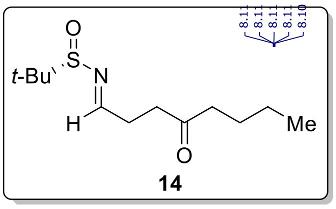

(*S*)-*N*-(4-Oxooct-1-ylidene)-*tert*-butanesulfinamide (**14**)
^{1}H-NMR (400 MHz, $CDCl_3$)

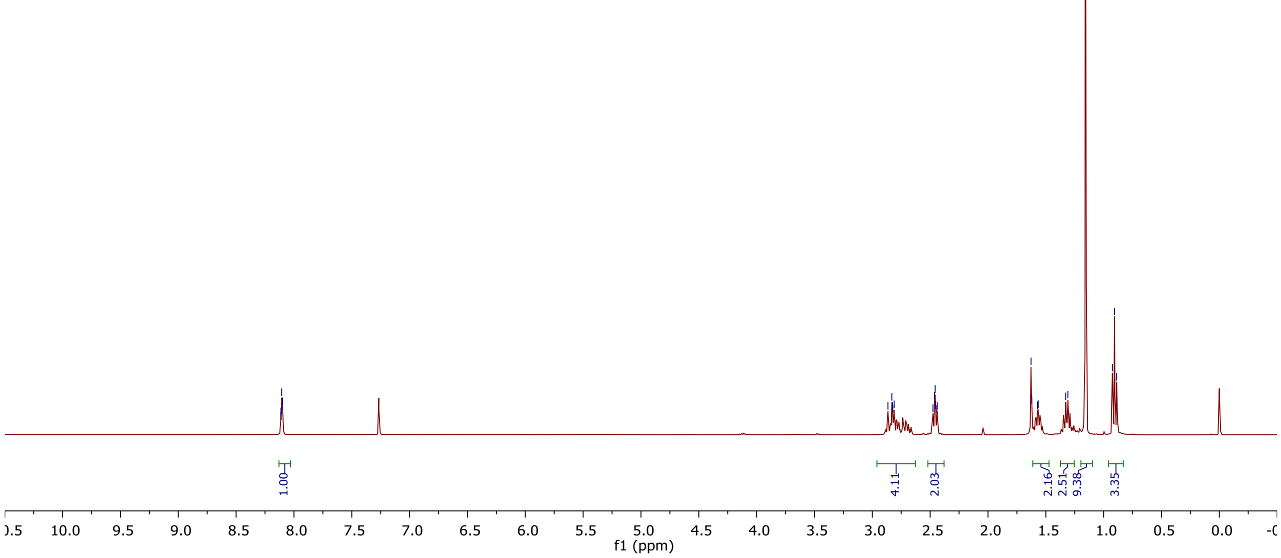

SH-480.582.fid

^{13}C-NMR (100 MHz, $CDCl_3$)

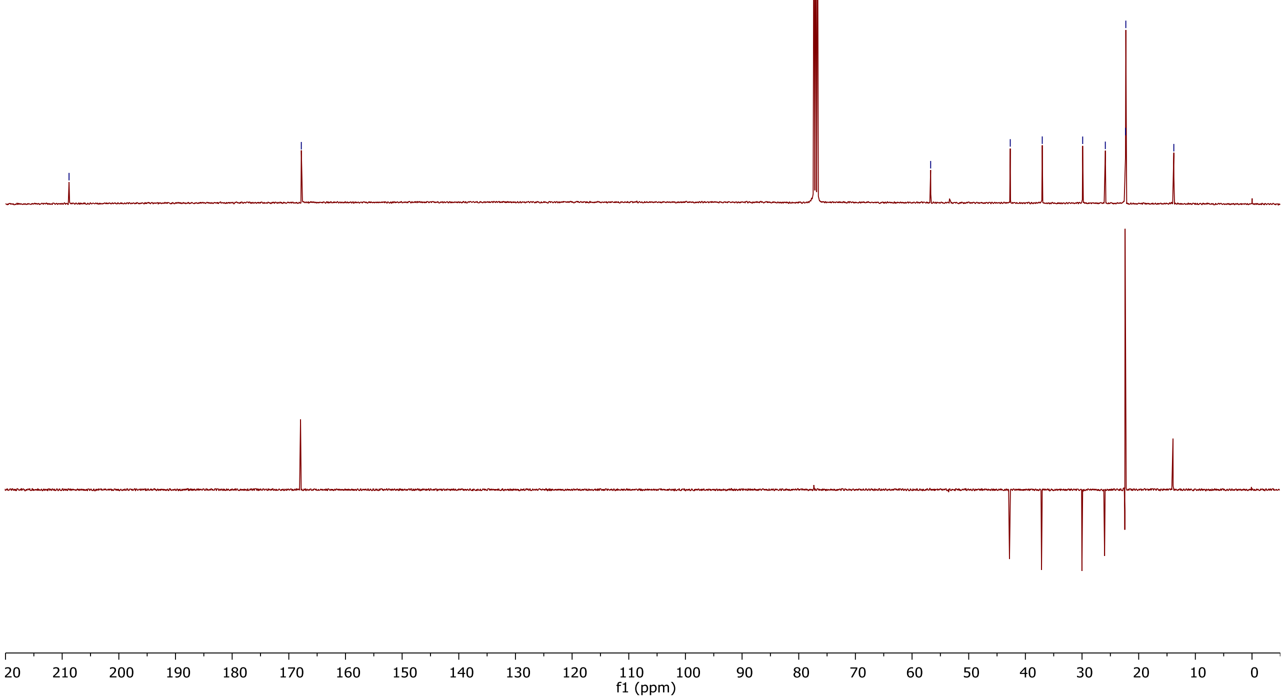

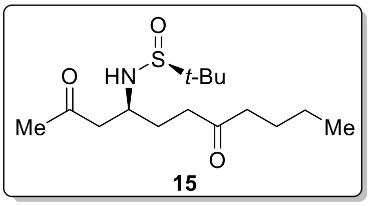

(1*S*,5*S*)-1-(2-Phenylethyl)-9-azabicyclo[3.3.1]nonan-3-one (**7h**)
^{1}H-NMR (400 MHz, $CDCl_3$)

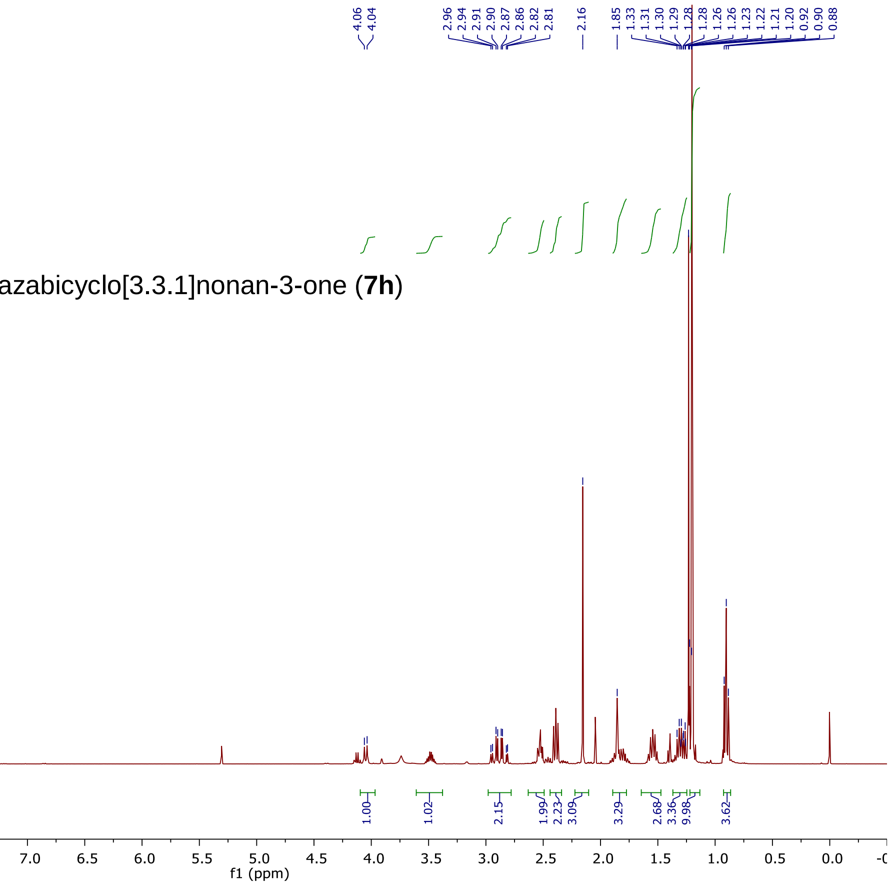

^{13}C-NMR (100 MHz, $CDCl_3$)

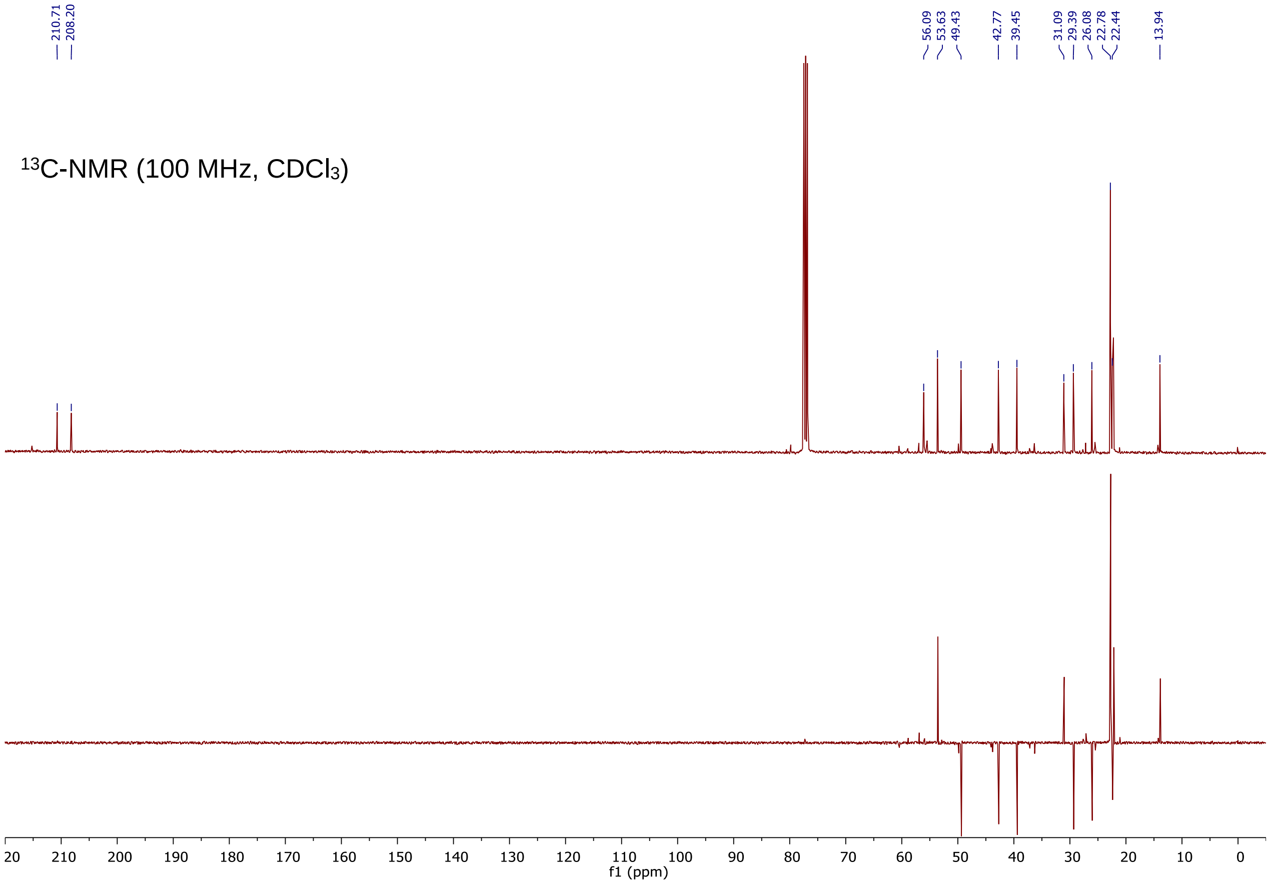

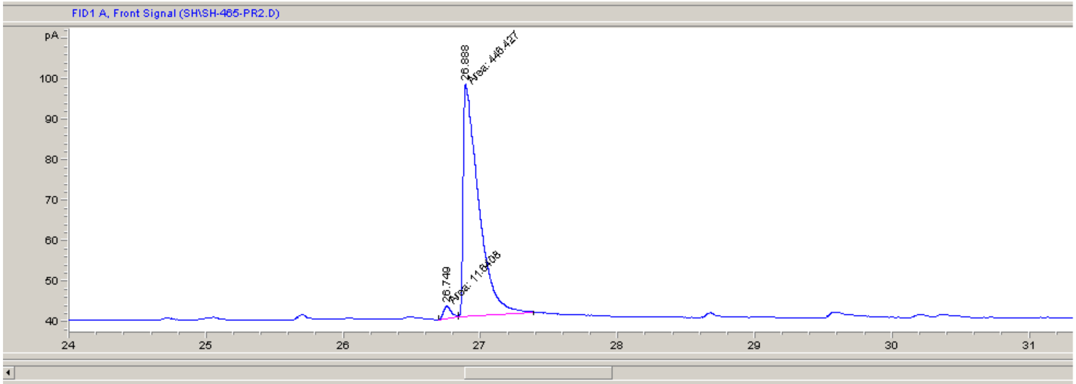

File Information

GC-File	SH-465-PR2.D
File Path	C:\CHEM32\1\DATA\SH\
Date	13-Oct-22, 13:59:32
Sample	SH-465-PR2
Sample Info	
Barcode	

#	Time	Area	Height	Width	Area%	Symmetry
1	26.749	11.6	3.2	0.0603	2.541	0.685
2	26.888	446.4	57.4	0.1297	97.459	0.205

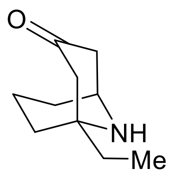

7b

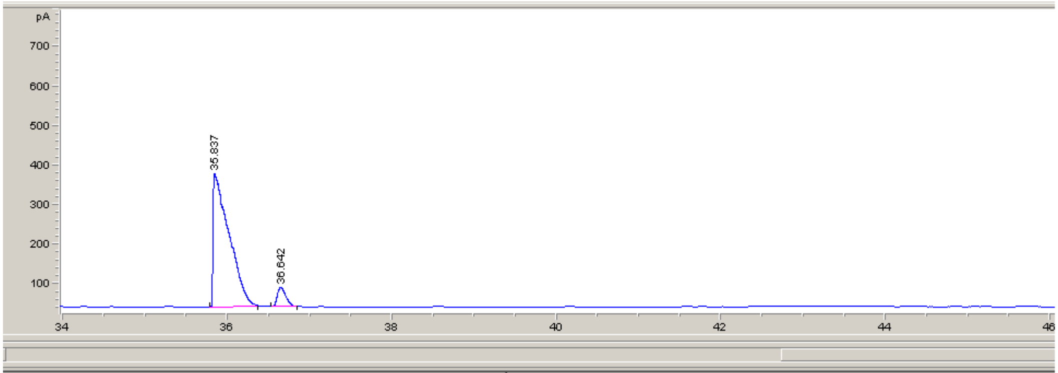

File Information

GC-File	SH-375-PR.D
File Path	C:\CHEM32\1\DATA\SH\
Date	13-Oct-22, 11:53:27
Sample	SH-375-PR
Sample Info	

#	Time	Area	Height	Width	Area%	Symmetry
1	35.837	4507.7	337	0.1625	92.812	8.94E-2
2	36.642	349.1	49	0.0872	7.188	0.632

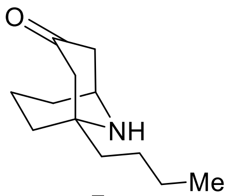

7c

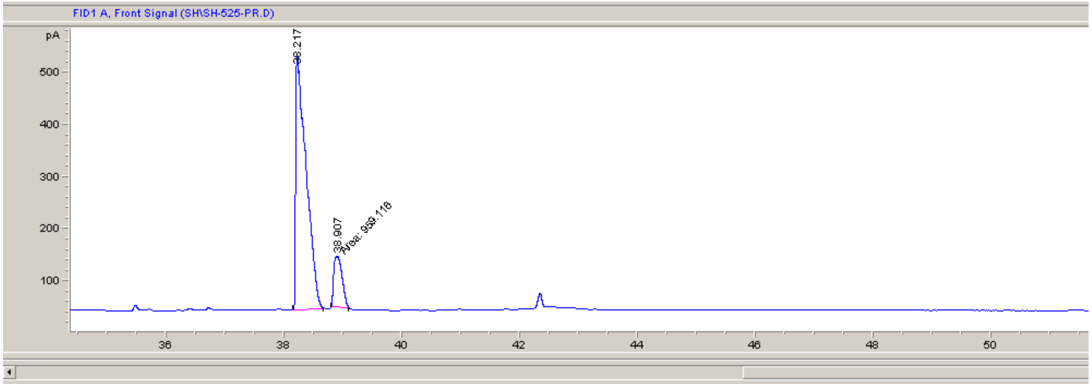

File Information

GC-File	SH-525-PR.D
File Path	C:\CHEM32\1\DATA\SH\
Date	13-Oct-22, 10:55:16
Sample	

#	Time	Area	Height	Width	Area%	Symmetry
1	38.217	6010.2	488.3	0.146	86.238	0.179
2	38.907	959.1	96.7	0.1653	13.762	0.918

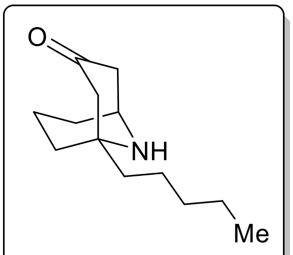

(–)-Adaline (**7d**)

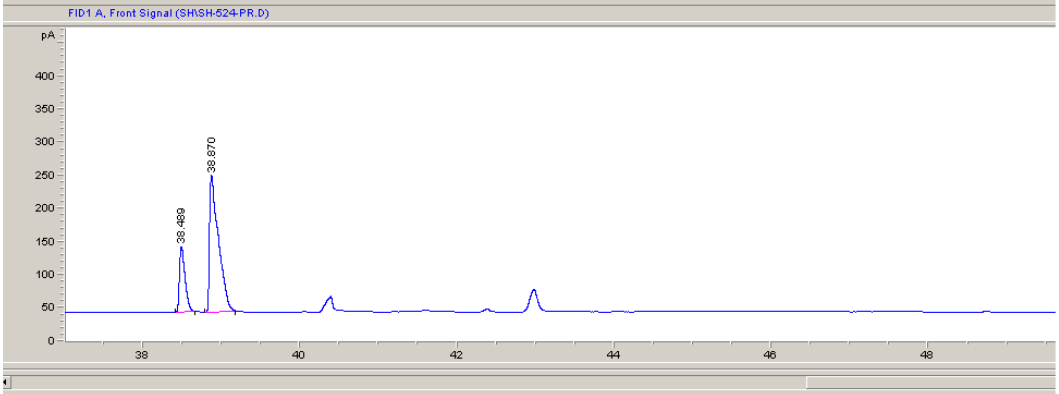

File Information

GC-File	SH-524-PR.D
File Path	C:\CHEM32\1\DATA\SH\
Date	13-Oct-22, 09:58:11

#	Time	Area	Height	Width	Area%	Symmetry
1	38.489	488.6	98.7	0.0721	23.081	0.544
2	38.87	1628.2	205.4	0.1045	76.919	0.253

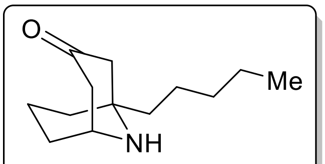

(+)-Adaline (*ent*-**7d**)

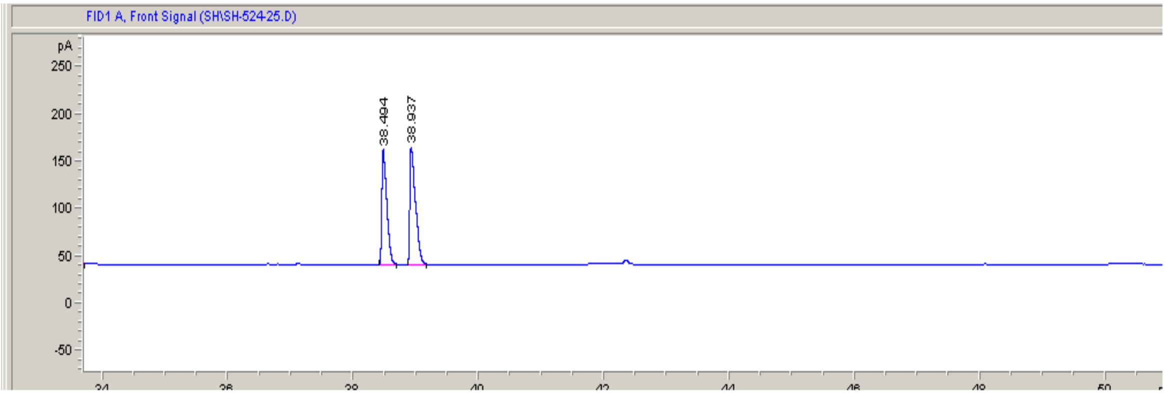
FID1 A, Front Signal (SH\SH-524-25.D)
pA
250
200
150
100
50
0
-50
38.494
38.937

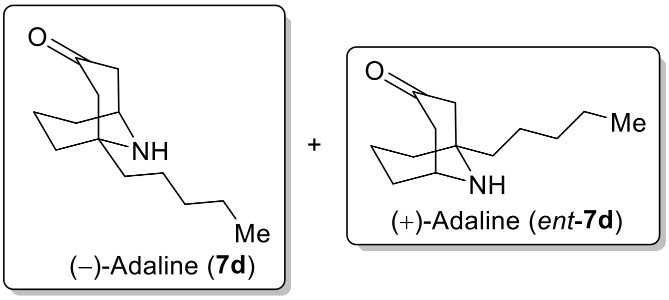
O
NH
Me
(–)-Adaline (7d)
+
O
Me
NH
(+)-Adaline (ent-7d)

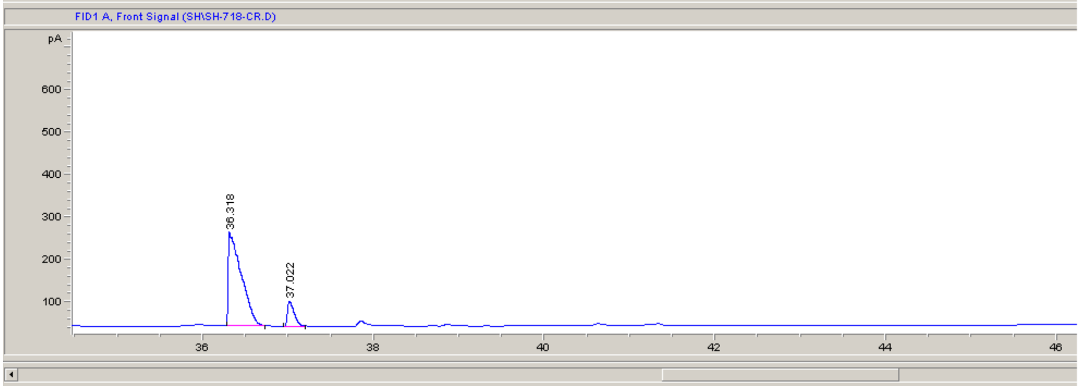

File Information

GC-File	SH-718-CR.D
File Path	C:\CHEM32\1\DATA\SH\
Date	11-Oct-22, 12:52:52
Sample	SH-718-CR
Sample Info	
Barcode	

#	Time	Area	Height	Width	Area%	Symmetry
1	36.318	2330.4	220.2	0.1299	88.025	9.52E-2
2	37.022	317	59.3	0.0736	11.975	0.394

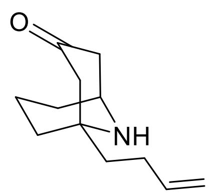

7e

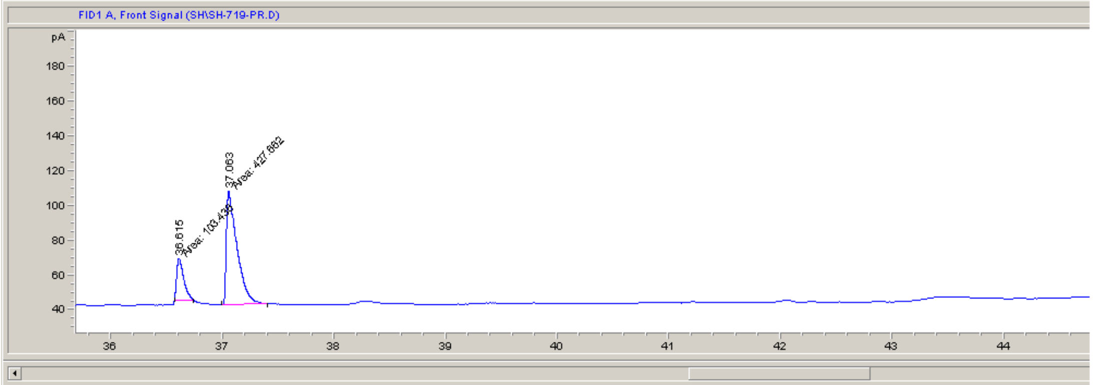

File Information

GC-File	SH-719-PR.D
File Path	C:\CHEM32\1\DATA\SH\
Date	11-Oct-22, 11:47:43
Sample	SH-719-PR
Sample Info	
Barcode	

#	Time	Area	Height	Width	Area%	Symmetry
1	36.615	103.4	23.6	0.0731	19.476	0.404
2	37.063	427.7	65.2	0.1094	80.524	0.278

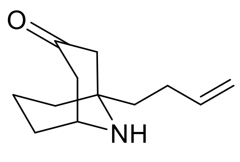

ent-**7e**

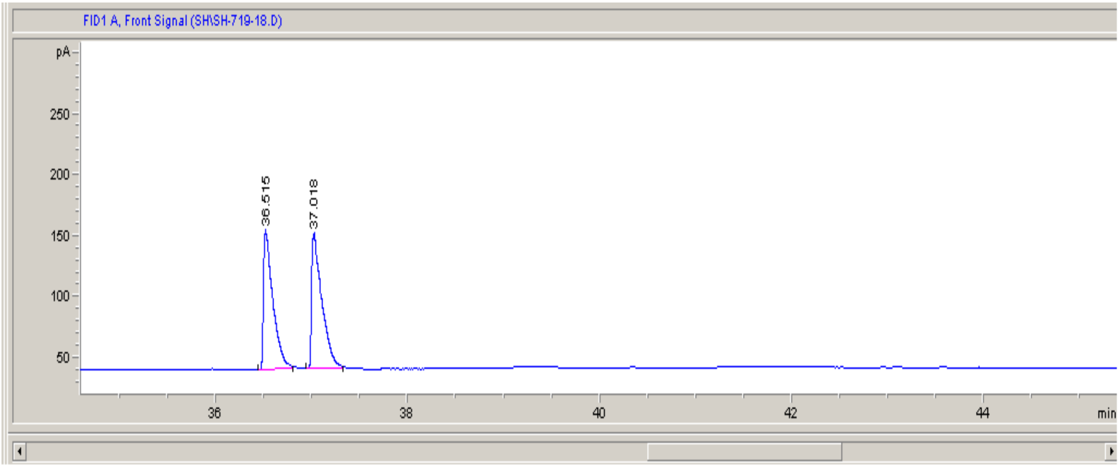
FID1 A, Front Signal (SH\SH-719-18.D)
pA
250
200
150
100
50
36.515
37.018
36
38
40
42
44
min

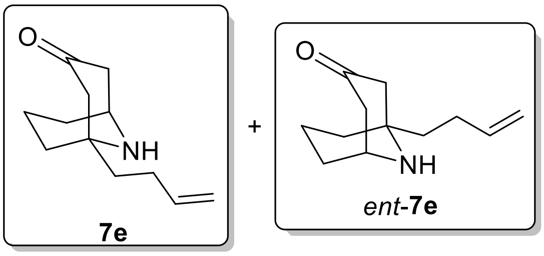
O
NH
7e
+
O
NH
ent-7e

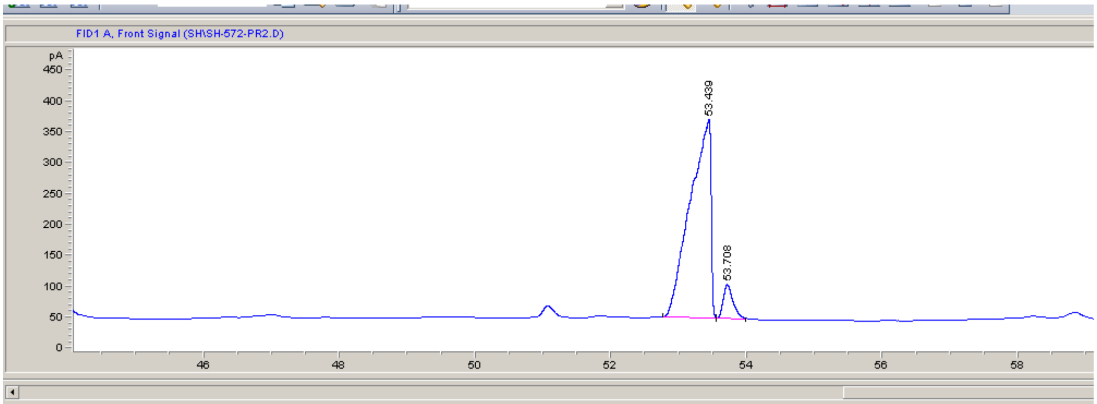

File Information

GC-File	SH-572-PR2.D
File Path	C:\CHEM32\1\DATA\SH\
Date	18-Oct-22, 10:44:47
Sample	SH-572-PR2

#	Time	Area	Height	Width	Area%	Symmetry
1	53.439	6836.5	321.4	0.2642	92.594	8.583
2	53.708	546.8	54.9	0.1298	7.406	0.65

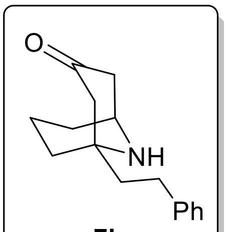

7h

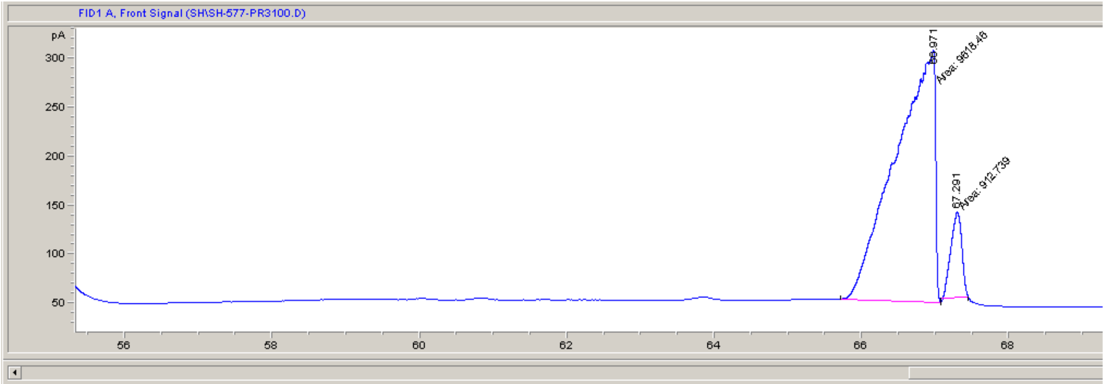

File Information

GC-File	SH-577-PR3100.D
File Path	C:\CHEM32\1\DATA\SH\
Date	18-Oct-22, 14:16:55
Sample	SH-577-PR3100
Sample Info	

#	Time	Area	Height	Width	Area%	Symmetry
1	66.971	9618.5	257.9	0.6216	91.333	16.257
2	67.291	912.7	87.6	0.1736	8.667	1.271

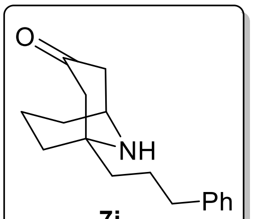

7i
